# Mutations in the Mitochondrial Methionyl-tRNA Synthetase Cause a Neurodegenerative Phenotype in Flies and a Recessive Ataxia (ARSAL) in Humans

**DOI:** 10.1371/journal.pbio.1001288

**Published:** 2012-03-20

**Authors:** Vafa Bayat, Isabelle Thiffault, Manish Jaiswal, Martine Tétreault, Taraka Donti, Florin Sasarman, Geneviève Bernard, Julie Demers-Lamarche, Marie-Josée Dicaire, Jean Mathieu, Michel Vanasse, Jean-Pierre Bouchard, Marie-France Rioux, Charles M. Lourenco, Zhihong Li, Claire Haueter, Eric A. Shoubridge, Brett H. Graham, Bernard Brais, Hugo J. Bellen

**Affiliations:** 1 Program in Developmental Biology, Baylor College of Medicine, Houston, Texas, United States of America; 2 Medical Scientist Training Program, Baylor College of Medicine, Houston, Texas, United States of America; 3 Laboratoire de Neurogénétique de la Motricité, Centre de Recherche du Centre Hospitalier de l'Université de Montréal, Montréal, Québec, Canada; 4 Department of Human Genetics, Montreal Neurological Institute–McGill University, Montréal, Québec, Canada; 5 Department of Molecular and Human Genetics, Baylor College of Medicine, Houston, Texas, United States of America; 6 Clinique des Maladies Neuromusculaires, Centre de Santé et de Services Sociaux de Jonquière, Saguenay, Québec, Canada; 7 Clinique des Maladies Neuromusculaires, Centre Hospitalier Universitaire Sainte-Justine, Montréal, Québec, Canada; 8 Service de Neurologie, Centre Hospitalier Affilié Universitaire de Québec–Hôpital de l'Enfant-Jésus, Université Laval, Québec, Québec, Canada; 9 Service de Neurologie, Centre hospitalier de l'Université de Sherbrooke, Sherbrooke, Québec, Canada; 10 Department of Medical Genetics, University of São Paulo, Ribeirao Preto–São Paulo, Brazil; 11 Howard Hughes Medical Institute, Baylor College of Medicine, Houston, Texas, United States of America; 12 Department of Neuroscience, Baylor College of Medicine, Houston, Texas, United States of America; 13 Jan and Dan Duncan Neurological Research Institute, Baylor College of Medicine, Houston, Texas, United States of America; St. Jude Children's Research Hospital, United States of America

## Abstract

An increasing number of genes required for mitochondrial biogenesis, dynamics, or function have been found to be mutated in metabolic disorders and neurological diseases such as Leigh Syndrome. In a forward genetic screen to identify genes required for neuronal function and survival in *Drosophila* photoreceptor neurons, we have identified mutations in the mitochondrial methionyl-tRNA synthetase, *Aats-met*, the homologue of human *MARS2*. The fly mutants exhibit age-dependent degeneration of photoreceptors, shortened lifespan, and reduced cell proliferation in epithelial tissues. We further observed that these mutants display defects in oxidative phosphorylation, increased Reactive Oxygen Species (ROS), and an upregulated mitochondrial Unfolded Protein Response. With the aid of this knowledge, we identified *MARS2* to be mutated in Autosomal Recessive Spastic Ataxia with Leukoencephalopathy (ARSAL) patients. We uncovered complex rearrangements in the *MARS2* gene in all ARSAL patients. Analysis of patient cells revealed decreased levels of MARS2 protein and a reduced rate of mitochondrial protein synthesis. Patient cells also exhibited reduced Complex I activity, increased ROS, and a slower cell proliferation rate, similar to *Drosophila Aats-met* mutants.

## Abstract 2

The study of *Drosophila* neurodegenerative mutants combined with genetic and biochemical analyses lead to the identification of multiple complex mutations in 60 patients with a novel form of ataxia/leukoencephalopathy.

## Author Summary

Neurodegenerative diseases, as a group, are relatively common and often devastating to those who suffer from them. Key insights are emerging from the study of homologues of identified human disease-causing genes in model organisms such as fruit flies, worms, and mice. In this study, we used the fruit fly to identify novel neurodegeneration-causing mutations and identified the *Aats-met* gene, whose protein product is involved in mitochondrial translation. We found that mutations in this gene cause neurodegeneration, impaired mitochondrial activity, and elevated oxidative stress. We were able to attenuate these defects with antioxidants like Vitamin E. We also determined that unusual duplications in the homologous human gene, *MARS2*, were responsible for a novel type of progressive ataxia found in some French Canadian families. Cells taken from these patients have many of the characteristic defects observed in flies, showing that the fly mutants can be used to further explore disease mechanisms and test potential treatments.

## Introduction

A number of neurological diseases are associated with mitochondrial dysfunction. For example, mutations in the mitochondrial genome have been found in a wide range of disorders including Leber's Hereditary Optic Neuropathy (LHON), Neuropathy, Ataxia and Retinitis Pigmentosa (NARP), Mitochondrial myopathy, Encephalopathy, Lactic Acidosis and Stroke (MELAS), Myoclonic Epilepsy associated with Ragged Red Fibers (MERRF), Nonsyndromic Sensorineural Deafness (NSSD), and Kearns-Sayre Syndrome [1],[2]. All of these disorders cause some dysfunction of the nervous system. Aside from these mitochondrially encoded genes, there is a growing list of mitochondria-targeted nuclear genes that when mutated cause diseases. These include (1) components of the respiratory chain/assembly factors [3],[4], (2) genes required for mtDNA maintenance/replication [5],[6], (3) genes that regulate dNTP pools [7], (4) genes that regulate mitochondrial morphology/cellular trafficking [8],[9], and (5) genes involved in mtDNA transcription and translation [10].

Mitochondria are critical for energy production and are intricately linked to numerous aspects of cellular function. For example, cell proliferation defects have been reported for several mitochondrial fly mutants [11],[12]. It has been proposed that Complex I disruption results in reduced cell proliferation caused by the buildup of Reactive Oxygen Species (ROS). ROS are short-lived oxygen radicals that are produced at low levels as a result of impaired electron transport. These ROS can react with proteins, lipids, and DNA resulting in major damage to the cell and its mitochondria [13].

Studies in *Drosophila* have provided insight into the function of numerous human disease genes [14]. Indeed, work on the fly homologue of the then newly discovered *PARK2* gene responsible for Autosomal Recessive Juvenile Parkinson's Disease (OMIM #600116) [15] provided compelling evidence that *parkin* mutations result in mitochondrial dysfunction and oxidative stress [16],[17],[18], work that was subsequently confirmed in human cells [19],[20]. Forward genetic screens have also been carried out to isolate genes that cause a neurodegenerative phenotype [21],[22]. These forward genetic screens may allow us to identify novel genes and help us understand the cellular mechanisms required for neuronal survival. For example, the gene *nmnat*, whose loss has a strong neurodegenerative phenotype, encodes an important neuroprotective protein that may act as a chaperone [23],[24]. Interestingly, one of its orthologues in mice has been shown to confer significant neuroprotective effects in several disease models [25].

We decided to reassess the phenotypes of numerous mutants that were isolated in a mosaic eye screen in which we screened for defective electroretinograms (ERGs) in mutant photoreceptors on chromosome arm 3R [24],[26],[27]. Here we report the isolation and characterization of the *Drosophila* mitochondrial gene *Aats-met* (*Aminoacyl-tRNA synthetase-methionine*, NP_650348.1). We show that a partial loss of *Aats-met* results in mitochondrial dysfunction and causes a severe and progressive neurodegenerative phenotype. We further show that rearrangements in its human homologue, *MARS2* (Methionyl Aminoacyl-tRNA Synthetase 2, NP_612404.1), are responsible for a human neurodegenerative disease named ARSAL, for Autosomal Recessive Spastic Ataxia with Leukoencephalopathy, or Spastic Ataxia type 3 (SPAX3, OMIM #611390) [28].

## Results

### Isolation of the Fly Mitochondrial *Aats-met*

**Figure 1:**
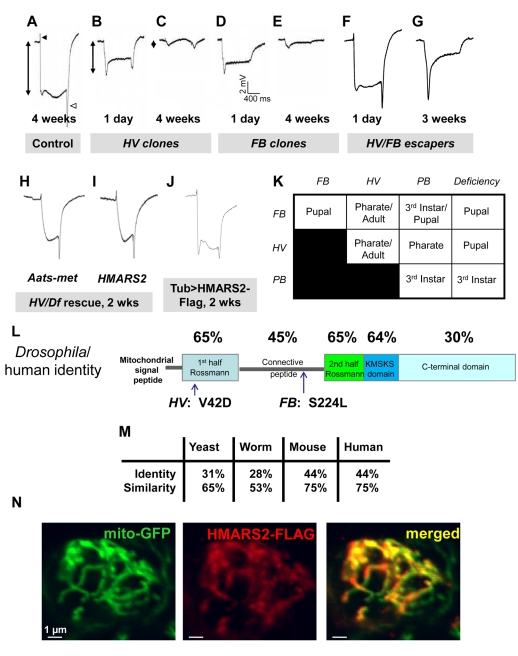
Identification/mapping of the *Aats-met* gene. (A) ERG of the control (*y w*; *FRT82B iso*). The black and white arrowheads indicate the “on” and “off” transients, respectively. The double-pointed arrow indicates the amplitude. (B–C) ERGs of homozygous *HV* clone-containing flies at 1 d and 4 wk after eclosion. (D–E) ERGs of homozygous *FB* clone-containing flies at 1 d and 4 wk after eclosion. (F) ERG of a 1-d-old *HV/FB* escaper. (G) ERG of a 3-wk-old *HV/FB* escaper. (H) ERG of a 2-wk-old *HV/Df* fly rescued with *actin-Gal4* and *UAS-Aats-met*. (I) ERG of a 2-wk-old *HV/Df* fly rescued with *actin-Gal4* and *UAS-HMARS2*. (J) ERG of a 2-wk-old otherwise wild-type fly expressing HMARS2-FLAG driven by tub-Gal4. (K) Lethal stages of homozygous and transheretozygous allelic combinations reveal an allelic series: *Df>PB>FB>HV*. (L) The Aats-met protein's predicted domains are shown (drawn to scale), with position of mutations and percentage identity compared to human MARS2 shown. (M) The *Drosophila Aats-met* gene is homologous to the mitochondrial methionyl-tRNA synthetase genes of *S. cerevisiae*, *C. elegans*, *M. musculus*, and *H. sapiens*. (N) Colocalization of the Flag-tagged human MARS2 protein with Mito-GFP in the cell body of a neuron in the ventral nerve cord, driven by the D42-Gal4 driver, is shown.

We reexamined a collection of lethal mutants generated on chromosome 3R to identify mutations that cause a degenerative phenotype [26]. We induced large clones of homozygous mutant tissue in the eyes using the ey-FLP system and screened for flies with aberrant ERGs that significantly worsen with age as a readout for degeneration of photoreceptors [29]. As shown in Figure 1, we isolated a lethal complementation group consisting of two alleles, *HV* and *FB*. Control flies exhibit an “on” transient (black arrowhead) upon a flash of light (Figure 1A). A change in potential ensues (arrow), which is followed by an “off” transient (white arrowhead) when the light is switched off. The *HV* and *FB* mutants produced ERGs with significantly reduced amplitudes (double-headed arrow) (Figure 1B,D), suggesting a defect in phototransduction and synaptic transmission. As the flies age, the ERGs exhibit gradually smaller amplitudes in response to light (Figure 1C,E). A less severe genetic combination of alleles that produces adult flies (see below), *HV/FB*, have normal ERGs at 1 d of age, while 3-wk-old animals (Figure 1F,G) have severely affected ERGs.

To map the *HV* and *FB* mutations we turned to meiotic recombination mapping with *P*-element lines [30] and deficiency mapping (Figure S1A–B). This pinpointed a 120 Kb region with 18 candidate genes. One lethal mutation, a *piggyBac* (*PB*) transposon insertion [31] in an intron of the *Aats-met* gene (*Aats-met^c00449^*), failed to complement the lethality of the *FB* allele (Figures 1K, S1B). Sequencing revealed that *HV* and *FB* affect the *Aats-met* gene: *HV* carries a c.125T>A predicted to result in the missense mutation p.V42D, whereas *FB* carries a c.671C>T predicted to result in the missense mutation p.S224L (Figure 1L). *Aats-met* encodes the uncharacterized *Drosophila* mitochondrial methionyl-tRNA synthetase, with 44% identity and 75% similarity to its human orthologue *MARS2* (Figure 1L,M) [32]. Complementation tests with the three alleles and a deficiency (*Df(3R)Exel7321*) indicate the following allelic series: *Df>PB>FB>HV*. Flies homozygous for *HV* or transheterozygous for *HV* and *FB* are semi-viable, although they exhibit reduced lifespans (see below). To demonstrate that the phenotypes associated with the mutations are indeed caused by a defective *Aats-met* gene, we ubiquitously expressed the *Drosophila Aats-met* and human *MARS2* cDNAs using the Gal4/UAS system in mutant backgrounds [33]. The fly and human cDNAs rescued the lethality associated with *FB/Df* and *HV/Df*, the strongest allelic combinations. Note that overexpression of these cDNAs in a wild-type background, ubiquitously or only in the eye, results in a wild-type ERG phenotype (Figure 1J). Moreover, the ERGs of aged *HV/Df* rescued flies are normal (compare Figure 1C with 1H–I), demonstrating that the mutations in *Aats-met* are indeed responsible for the lethality and ERG defects. These data also indicate that *MARS2* and *Aats-met* are homologous genes as both rescue the *Aats-met* mutants. We also Flag-tagged the human *MARS2* construct at the C-terminus and performed colocalization experiments with the mitochondrial reporter mito-GFP protein [34] in mitochondria of Central Nervous System neurons of 3^rd^ instar larvae (Figure 1N). Both proteins co-localize, indicating that MARS2 is indeed a mitochondrial protein.

### Loss of *Aats-met* in the Eye Results in Retinal Degeneration

**Figure 2:**
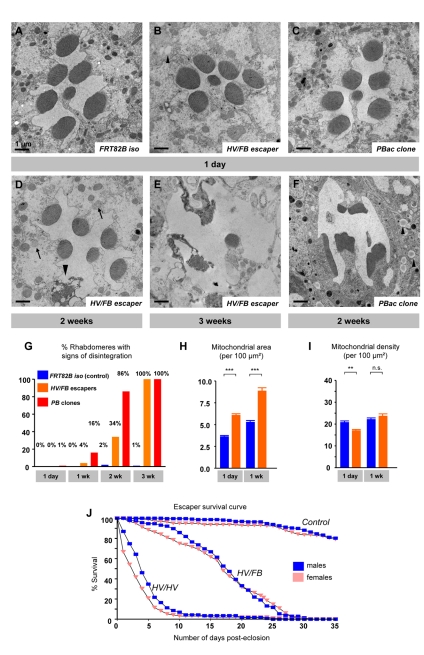
Retinal degeneration and lifespan of *Aats-met* mutants. (A) TEM of a single ommatidium from a control 1-d-old fly eye, showing the characteristic seven dark rhabdomeres in the center. (B) TEM of a single ommatidium from the eye of a 1-d-old *HV/FB* escaper fly, showing no obvious defects. (C) TEM of the eye of a 1-d-old fly containing homozygous clones of a *PB* allele. (D) TEM of the eye of a 2-wk-old *HV/FB* escaper fly, showing the beginning of a neurodegenerative process, with a degenerating rhabdomere (arrowhead) and enlarged mitochondria (arrow). (E) TEM of the eye of a 3-wk-old escaper. (F) A neurodegenerative process is evident in clones of the *PB* allele in a 2-wk-old fly. Arrows indicate lipid droplets in pigment cells (arrowheads). (G) Quantification of 100 retinal photoreceptor rhabdomeres for the control, *HV/FB* escapers, and *PB* clone-containing mutants at different ages. (H) Quantification of the total mitochondrial area as a percentage of the retinal area: *HV/FB* mutants clearly have a higher mitochondrial content. (I) Quantification of average mitochondrial size, showing the mitochondrial number of the *HV/FB* mutant retinas (*n* = 50). (J) Graph showing the shortened lifespans of 100–200 *HV/FB* and *HV/HV* escapers of each gender compared to controls, with males denoted in blue and females in pink. Scale bars: 1 µm.

To assess whether a worsening of the ERG phenotype is due to progressive degeneration of photoreceptor neurons (PRs) in *Aats-met* mutant retina, we performed Transmission Electron Microscopy (TEM) of the retinas of flies of different ages. We focused our analysis on transheterozygous escapers (*HV/FB*) and clones of the *PB* allele. Both have normal ERGs (Figure 1F), with no obvious developmental defects, and possess the correct number of photoreceptors per ommatidium in 1-d-old animals (Figure 2A–C). They display no defects in their rhabdomeres, and the overall appearance of the PRs also appears normal. As shown in Figure 2D–E and 2G, the PRs and support cells (glia) progressively degenerate. By 2 wk of age, the PRs of *HV/FB* animals display more severe phenotypes, and some PRs are vacuolated (arrowhead, Figure 2D). By 3 wk of age, most PRs are severely affected and many organelles are barely recognizable (Figure 2E,G). Similarly, in mutant clones of the *piggyBac* (*PB*), PRs are mostly normal at day 1 (Figure 2C) and become severely affected by 2 wk of age (Figure 2F). In summary, different mutations cause a severe progressive degeneration of PRs and glia.

A careful quantitative analysis of the TEM micrographs revealed some subtle defects in young animals. Indeed, the total mitochondrial area in mutant PRs is greater in 1-d- and 1-wk-old animals (2-wk-old animals were too severely affected to quantify) (Figure 2H). In addition, we also noted many grey spheres in the glia in mutants, indicating the presence of lipid droplets that are not observed in wild-type animals (black arrowhead, Figure 2B,F). That these are indeed lipid droplets was confirmed with toluidine blue staining (red arrows in Figure S1E–F), a possible indication of a fatty acid metabolism defect [35]. In summary, the electrophysiological and ultrastructural features indicate that the mutant photoreceptor neurons undergo progressive degeneration.

### Loss of *Aats-met* Results in Reduced Lifespan and Muscle Degeneration

**Figure 3:**
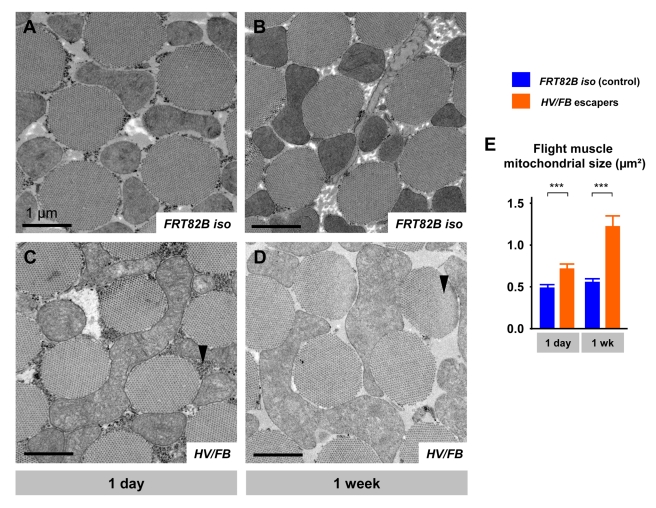
TEM of indirect flight muscle. (A) TEM micrograph of 1-d-old control (*FRT82B* isogenized) flight muscle, with its characteristic myofibers surrounded by mitochondria and small glycogen granules. (B) Micrograph of 1-wk-old control muscle. (C) Micrograph of 1-d-old *HV/FB* escaper, with much larger mitochondria with poor cristae structure, and a high density of granules compared to control (arrowhead). (D) 1-wk-old escaper flight muscle, with a similar but more severe mitochondrial phenotype and a complete absence of granules. Myofibril degeneration is highlighted by the arrowhead. (E) Quantification of the average mitochondrial size between control (blue) and *HV/FB* (orange) escaper flight muscle, showing much larger mitochondria present in the mutants. Scale bars: 1 µm.

*HV/FB* and *HV/HV* escapers are morphologically normal. They feed, walk, and mate, suggesting that their development and basic physiological features are relatively normal. They, however, have much shorter lifespans than wild-type flies (Figure 2J) and are unable to fly. In light of their inability to fly and shortened lifespans, we examined the indirect flight muscles of these flies. Interestingly, the myofibrils seem intact at 1 d of age (Figure 3A,C), but the mitochondria are clearly aberrant: they are larger than normal (Figure 3C–E). In 1-wk-old *HV/FB* flies, the myofibrils display defects (arrowhead in Figure 3D), and the mitochondria are very large (Figure 3D–E). At 2 wk of age the muscle is too fragmented to take TEM images. Hence, partial loss-of-function mutations in *Aats-met* impair longevity and mitochondrial morphology.

### Cell Proliferation Is Impaired in *Aats-met* Mutants

**Figure 4:**
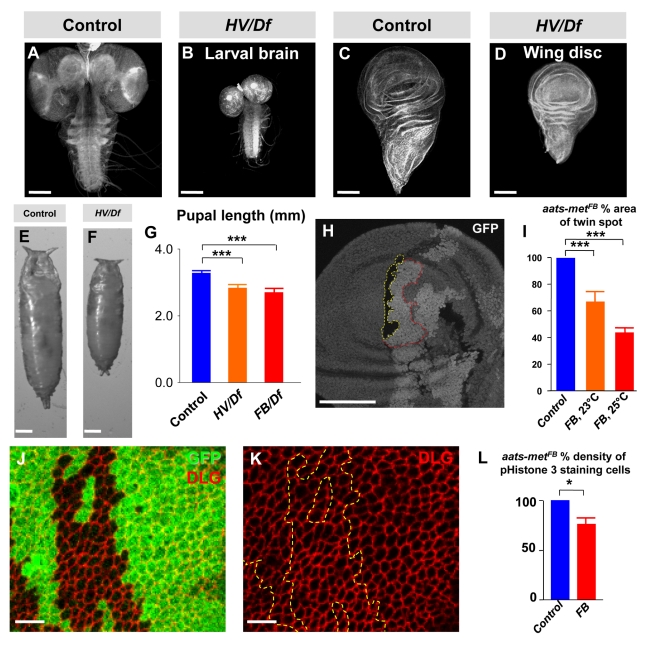
*Aats-met* mutants have reduced cell proliferation. (A–B) Brains of late 3^rd^ instar control and *HV/Df* larvae stained with Rhodamine-Phalloidin. (C–D) Wing discs of a late 3^rd^ instar control and mutant larvae stained with Rhodamine-Phalloidin. (E–F) Control and mutant pupae are shown. (G) Quantification of pupal length is shown. (H) Wing disc containing wild-type (outlined in yellow) and mutant clones (outlined in red) are seen. (I) Wild-type clones are significantly larger than mutant clones, quantified in 16 to 20 pairs of clones. (J–K) Cells in mutant clones in wing discs, stained with anti-Dlg, to mark the cell membrane, are similar in size to wild-type cells. (L) PH3-staining cells in mutant versus neighboring heterozygous tissue is quantified for five wing discs, indicating that there is less cell proliferation in mutant clones. Data are mean ± s.e.m. Scale bars for (A–D) and (H) are 100 microns, (E–F) are 0.3 mm, and (J–K) are 5 microns.

We noted that *HV/Df* mutants die as late 3^rd^ instars or small pupae, possessing small imaginal discs and larval brains (Figures 1K, 4A–G). Despite their smaller size, mutant larval brains do not show any obvious differences in the immunostaining patterns and localization of neuronal and glial proteins like Elav, Bruchpilot, Fasciclin II, and Repo when compared to wild-type brains (unpublished data). Mutant cells exhibit a proliferative disadvantage when compared to wild-type cells as the mutant clones are significantly smaller than their wild-type twin spots in wing imaginal discs (Figure 4H–I). Moreover, anti-phosphoHistone 3 (PH3) staining, a mitotic cell marker, is decreased by 23% in mutant clones when compared to wild type clones in wing imaginal discs (Figures 4L and S2A), suggesting that cell proliferation is affected. However, cell growth does not seem to be significantly impaired based on staining against the cell membrane marker Dlg (Figure 4J–K). We also observed no difference in the number of apoptotic cells between wild-type and mutant clones based on Caspase 3 staining (Figure S2B–C), and ubiquitous overexpression of the anti-apoptotic protein P35 did not suppress the small larval brain phenotypes (Figure S2D–G). In summary, these data strongly indicate that *Aats-met* affects cell proliferation but not cell growth and apoptosis in non-neuronal cells.

### Upregulated UPR^mt^ in *Aats-met* Mutants

A mitochondria-specific stress response (UPR^mt^) induced by the overexpression of a misfolded mitochondrial matrix protein in mammalian cells has been described [36] and confirmed to be present in *C. elegans* [37]. In *C. elegans*, many of the RNAi constructs found to activate the UPR^mt^ correspond to mitochondrial translation factors [38]. Since *Aats-met/MARS2* is a mitochondrial translation factor, and since the highly conserved mitochondrial chaperone Hsp60 is a good reporter of the UPR^mt^ in *C. elegans*, we examined expression of Hsp60 [39]. We observe an elevation in Hsp60 levels in *Aats-met* mutant clones in the eye (Figure S3A–B) as well as in mutant clones in the wing imaginal discs (Figure S3C–D). To determine if the cytoplasmic UPR is affected, we carried out immunohistochemical stainings with BiP/Hsc3, which has been shown to be a reliable marker in flies for the cytoplasmic UPR [40],[41]. Unlike Hsp60, BiP/Hsc3 is not induced in mutant cells, indicating that the two UPR processes are uncoupled (Figure S3E–F).

### Aberrant Mitochondrial Respiration in *Aats-met* Mutants

**Figure 5:**
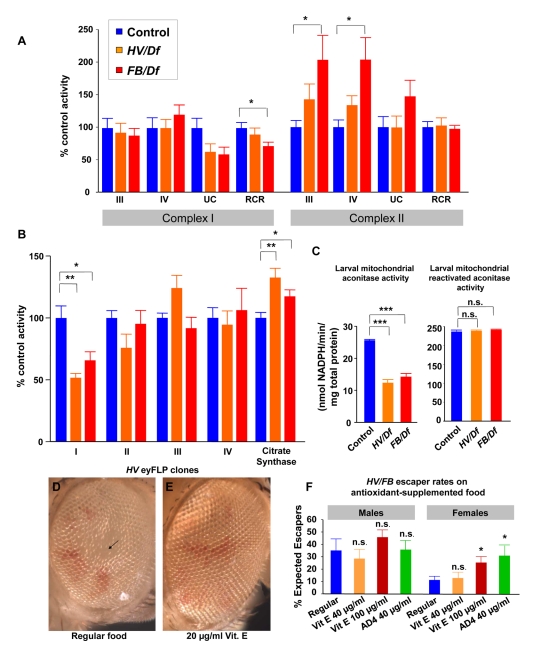
*Aats-met* mutants exhibit a complex I deficiency and phenotypes can be suppressed with antioxidants. (A) Polarography (measurement of substrate-dependent O_2_ consumption of isolated 3^rd^ instar larvae-derived mitochondria given needed substrates) was performed in the presence of Complex I–specific substrates or Complex II–specific substrate. State III is the ADP-stimulated oxygen consumption rate; state IV is the ADP-limited oxygen consumption rate; UC is the oxygen-consumption rate in the presence of an uncoupler; RCR is the Respiratory Control Ratio (state III rate/state IV rate). (B) Individual respiratory chain activities were measured from disrupted mitochondria. Mutant mitochondria exhibit partial deficiency of complex I as well as an increase in CS activity. Data are expressed as percentage control activity (mean ± s.e.m.). (C) Purified disrupted mitochondrial extracts from control 3^rd^ instar, *HV/Df*, and *FB/Df* larvae were quantified for aconitase activity, showing a significant decrease resulting from oxidation in the mutants. Treatment with reducing agent resulted in normal activity levels, indicating that the difference was not due to lower levels of aconitase but from increased oxidized aconitase. (D–E) *Aats-met^HV^* eyes often exhibit glossy areas in the middle of large clones (arrow). In addition, the eyes are typically smaller. With 20 µg/ml Vitamin E, there is significant improvement in eye morphology and size (*p*<0.001). (F) Mutant escaper rates are increased for females supplemented with antioxidants. Male escaper rates are already high, even without antioxidants. Three different drug supplementation regimens were used. For the female escaper rate, the last two drug regimens produced a significant improvement. Data are mean ± s.e.m.

To assess the functional consequence of mutations in *Aats-met* on oxidative phosphorylation, the rate of oxygen consumption of intact mutant mitochondria was measured *in vitro* by performing polarography [42]. In the presence of the Complex I–specific oxidizable substrates malate and glutamate, mutant mitochondria exhibit a decreased respiratory control ratio (RCR), the ratio of state III (ADP-stimulated O_2_ consumption rate) to state IV (ADP-limiting O_2_ consumption rate). The RCR for the most severe allelic combination (*FB/Df*) was significantly lower compared to control mitochondria, primarily due to a relative increase in the state IV rate, likely reflecting a partial uncoupling of oxidative phosphorylation in mutant mitochondria (Figure 5A). Interestingly, the oxygen consumption rates in the presence of the Complex II–specific oxidizable substrate succinate are increased for *Aats-met* mutant (*FB/Df*) mitochondria compared to controls, while the RCRs remain preserved, possibly indicating a compensatory response (Figure 5A, Table S1). This is consistent with the finding in *C. elegans* of increased Complex II–dependent respiration activity when levels of various Complex I components are knocked down with RNAi [43]. Given that the mitochondrial genome encodes 13 polypeptides that are all components of the mitochondrial Electron Transport Chain (ETC) (Table S3), we investigated whether there is a respiratory chain deficiency. To directly assess the individual ETC complexes, enzyme activities of the individual respiratory chain complexes from purified and disrupted mitochondria were measured spectrophotometrically. We observed a significant decrease in Complex I activity (Figure 5B, Table S2). The partial deficiency of Complex I in mutant mitochondria is relatively mild given that 7 out of the 40 or more Complex I subunits are encoded in the mtDNA and are therefore dependent on mitochondrial protein translation (Table S3).

### Increased ROS and Suppression by Antioxidants

It has been proposed that high levels of ROS (primarily superoxide anion) because of aberrant Complex I activity results in reduced cell proliferation (Figure 4H–I), although low levels appear to promote proliferation [12],[44]. Hence, we hypothesized that the reduced cell proliferation in *Aats-met* mutants may be caused by elevated levels of ROS. Since mitochondrial aconitase activity is highly sensitive to ROS [45],[46], we measured aconitase activity normalized to total protein levels and found it to be greatly reduced (Figure 5C). Upon addition of a reducing agent, the aconitase activity is restored in the mutants, showing that aconitase is indeed more oxidized in the mutants than in the wild-type controls.

One of the mutant phenotypes associated with loss of *Aats-met* in the eye is very similar to the loss of *Pdsw*, which affects Complex I [12]. Clones of *Pdsw* in the eye cause a glossy patch and reduce the eye size. As shown in Figure 5D, *Aats-met* mutant clones exhibit similar phenotypes. We therefore tested if these phenotypes can be suppressed by antioxidants and supplemented with food with the lipophilic/cell-permeable Vitamin E (α-tocopherol) and water-soluble N-acetylcysteine amide (AD4) [47]. We scored the loss of the glossy patch and the number of ommatidia. As shown in Figure 5D–E, low levels of Vitamin E (20 µg/ml) significantly improved eye morphology and size (*p*<0.001). In addition, the percentage of adult female escapers of the genotype *HV/FB* able to eclose at room temperature increased significantly with antioxidants, although this was not observed in males (Figure 5F). Note that the doses of Vitamin E and AD4 used had no effect on wild-type eyes or eclosion rates (unpublished data).

### ARSAL Patients Carry Deletions and Duplications of the *MARS2* Locus

We noted that the human orthologue of *Aats-met*, *MARS2*, was located in a 3.33 Mb candidate interval on chromosome 2q33.1. Some of the authors of this manuscript had previously mapped a neurologic disease named ARSAL to this interval [28]. ARSAL is found in a large cohort of French-Canadian families and is an autosomal recessive spastic ataxia frequently associated with white matter changes as detected by MRI [28]. To examine this region closer, we generated Single Nucleotide Polymorphism (SNP) haplotypes using the 300K Illumina SNP-array on selected families. This documented the existence of three different disease carrier haplotypes in French-Canadian ARSAL cases (Figure S4). Recombination events within families established a minimum candidate interval of 579 Kb (rs16865262–rs7581202) (black bar in Figure S4), containing nine genes including *MARS2*. *MARS2* is a single exon gene that spans 3,528 bp of genomic DNA and encodes a 593 aa protein homologous to *Aats-met* [32]. Interestingly, no point mutations were uncovered by genomic or cDNA sequencing.

**Figure 6:**
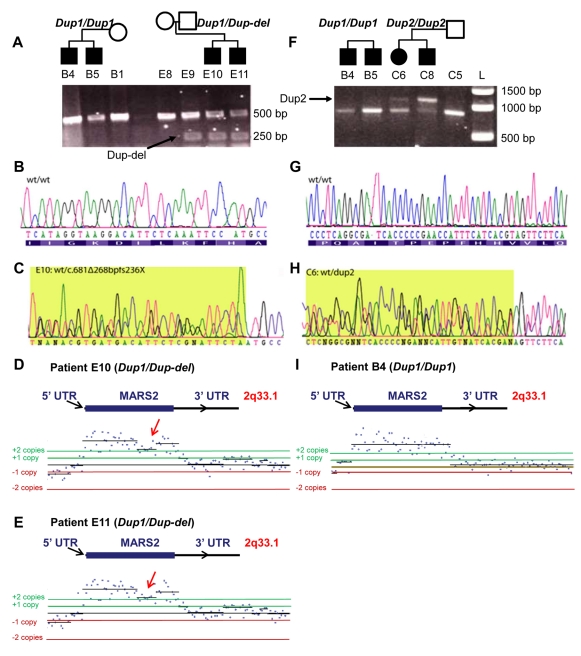
The human *MARS2* mutations. (A) PCR amplification products of *MARS2* encompassing a portion of the coding sequence revealed the presence of a 268 bp deletion mutation segregating in ARSAL Family E but not in Family B. This truncated product is indicated by an arrow. The normal PCR product is around 500 bp. Segregation of the deletion is shown in Family E; brothers E10 and E11 carry the mutation. Their unaffected father E9 is also a carrier. The determined genotypes for the patients shown (summarized in Table S5 for all patients) are shown above the PCR bands. (B) Wild-type sequence of *MARS2* PCR products. (C) DNA sequencing of the deletion (c.681Δ268bpfx236X). (D–E) Nonrecurrent rearrangements involving the *MARS2* gene was confirmed by the oligonucleotide custom aCGH. In patients E10 and E11, the array discriminated the presence of the duplication as well as the deletion (see arrows) as depicted by the lower band detecting only one additional copy. (F) PCR amplification products of *MARS2* encompassing the coding sequence revealed the presence of a ∼300 bp insertion mutation segregating in ARSAL family members C6 and C8 but not in Family B. This larger amplification product is indicated by an arrow. The normal amplicon size is about 800 bp. C5 is the unaffected father of C6 and C8 and also carries the mutation. (G) Wild-type sequence of *MARS2*. (H) DNA sequencing of the heterozygous case C6 corresponding to the insertion revealed parts of the *MARS2* duplication mutation. Rearrangement was confirmed by oligonucleotide custom aCGH. Note that the array data of C6, a compound heterozygote (*Dup2/Dup2*), demonstrates the presence of a potentially larger duplication while not showing the 300 bp insertion, the array not having been designed to include its sequence. (I) In homozygous patient B4 (*Dup1/Dup1*), the array suggests that the duplication has identical distal and proximal breakpoint junctions with the other ARSAL cases.

The first mutation was identified by PCR in Family E and consists of a 268 bp deletion predicted to lead to a premature STOP codon at position 236 (c.681Δ268bpfs236X), referred to subsequently as Dup-Del (Figure 6A). This deletion was confirmed by sequencing in nine patients from different families (Tables S4 and S5). As shown in Figure 6A, PCR amplification of *MARS2* encompassing the first third of the coding sequence revealed the presence of a deleted fragment that segregates in ARSAL Family E (arrow, E9, E10, E11) and can also be seen in the father (E9), who is an unaffected carrier. This deleted fragment is not observed in the mother (E8) and in Family B members, who possess a different type of mutation in the *MARS2* gene (see below). The wild-type sequence of the *MARS2* PCR products (Figure 6B) and DNA sequencing of the amplicon of compound heterozygous case E10 documents the deletion (compare Figure 6C to 6B). This mutation was confirmed by oligonucleotide custom array Comparative Genomic Hybridization (aCGH), as discussed below. Interestingly, the deletion is part of a complex duplication of *MARS2* in these patients (see below). In affected brothers E10 and E11, the aCGH discriminated the presence of a duplication (black lines/dots above the +2 copies green line in Figure 6D) in both patients as well as a deletion (red arrows in Figure 6D–E, compare to Figure 6I).

**Figure 7:**
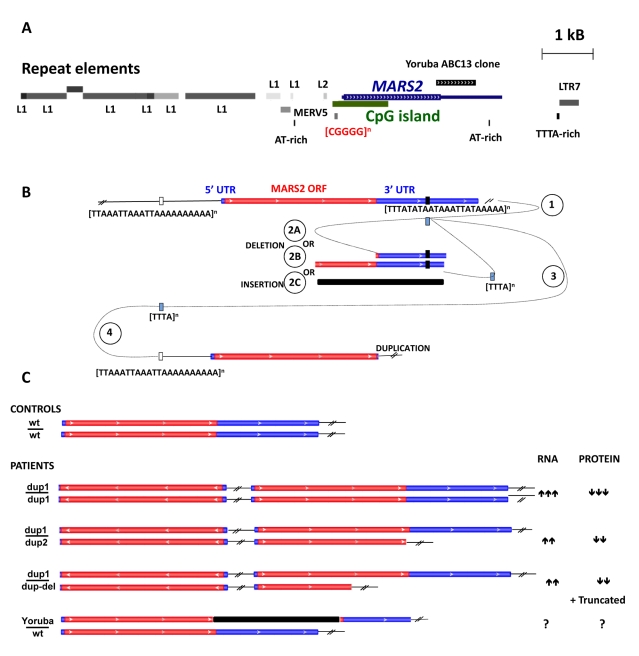
Schematic representation of the MARS2 region and ARSAL mutations. (A) Schematic representation of the chromosome 2q33.1 locus containing the mitochondrial methionyl-tRNA synthetase sequence (based on the UCSC genome browser). *MARS2* is an intronless gene located within the intronic sequence of a noncoding mRNA (BC021693). Its CpG island encompasses much of its coding sequence. Human Genome Structural Variation Project data show the insertion of a 726 bp discordant clone (ABC8_43216400 E17, Yoruba sample) containing a 276 bp LINE sequence (L2) within the coding sequence of the *MARS2* gene. DNA of this clone is depicted as a black box below the *MARS2* ideogram. Interestingly, the clone insertion fragment is located within the same distal junction breakpoint of ARSAL CNVs. (chr2: 198,280,073–198,280,860). No polymorphic CNV, structural variation, or segmental duplication have previously been reported on chromosome 2q33.1. Repeat elements are depicted as grey boxes. Using several combinations of primer pairs, genomic sequencing of carrier chromosomes allowed us to cover over 7 Kb and showed a partial deletion sequence at the 5′ region of *MARS2* and an insertion in the 3′ region. Sequencing and CGH-array data suggest that homologies among repeat elements are responsible for complex rearrangements accompanying the MARS2 duplications. (B) Illustration of the putative order and origin of the complex rearrangements found in the *MARS2* gene in ARSAL patients. The gene begins on the left (5′). The ORF is colored red and the UTRs blue. As mentioned above, the events share a common junctional sequence position, near the stop codon (black box). The presence of repetitive elements within MARS2 3′UTR and at the 5′ end is suggestive of a template-driven event (event (1) slippage or replication fork pause) that caused partial deletions or insertion (ABC8_43216400 E17, Yoruba sample) at the DNA lesion site (event (2A), (2B), or (2C)). We hypothesize that the complex genomic architecture that has similar sequence features may be able to form cruciform structures, suggesting that these events may be recurrent and stimulated by the abundance of AT-rich sequences around and within the *MARS2* gene (event (3)). The replication fork may have switched to another nearby homologous template consisting of short direct or inverted repeats (event (4)) resulting in the generation of duplication events, which could be advancing in either direction. Sequencing and CGH-array data suggest that homologies among repeat elements are responsible for the yielding of complex rearrangements accompanying the *MARS2* duplications, but we could not determine the orientation. (C) Illustration of the four predicted rearrangements of the *MARS2* region seen in ARSAL patients. The most common rearrangement is Duplication 1, in which two copies of *MARS2* are detected on each chromosome. The first one contains the entire coding and noncoding sequence, however the duplicated copy includes only the coding sequence. The brackets (//) refer to the fact that the duplication occurs at a distance from the endogenous *MARS2* gene, at least 15 Kb away, based upon our quantitative Southern data. Duplication 2 is very similar to the first one with the exception that the rearrangement includes a small deletion in the 3′UTR (caused by event 2A). The genomic structure of the third mutation (Duplication-Deletion) displays a large deletion of the *MARS2* coding region (referred by the event 2B) resulting in a truncated MARS2 protein. Quantitative experiments on both genomic and mRNA reveal a deletion rearrangement with partial duplication of the coding region of *MARS2*. A 726 bp discordant clone (ABC8_43216400 E17, Yoruba sample) containing a 276 bp LINE sequence (L2) within the coding sequence of the *MARS2* gene is reported in the UCSC track from the Human Genome Structural Variation Project data, though its impact on mRNA and protein is unknown.

Further evidence that mutations in *MARS2* were causative came from the identification of a 300 bp insert in the coding sequence that segregated within Family C (patients C6 and C8 in Figure 6F but not in Family B, which possesses a different mutation—see below). The insertion's sequence provided evidence of a complex 5′ mutation, since only a partial sequence of *MARS2* was revealed (Figure 6G,H). The presence of repetitive sequences at the 5′ end of *MARS2* combined with a 250 bp GC-rich sequence immediately 5′ of the ATG hampered *MARS2* full genomic sequencing. This region is 67% GC-rich and contains a 27 bp G/C stretch that is not polymorphic in controls ([CGGGG]^n^ in Figure 7A). The small size of the gene and the limited number of restriction sites prevented us from generating informative Southern blots to further investigate the breakpoints of rearrangements. Nevertheless, quantitative Southern Blot analysis using five additional restriction enzymes (*ApaI*, *NcoI*, *XhoI*, *KpnI*, and *HindIII*) confirmed the presence of the duplication (unpublished data). Based on our Southern blots, we conclude that the *MARS2* breakpoints are >15 Kb away from the wild-type copy of the *MARS2* gene. In summary, the presence of two mutations in the *MARS2* locus was documented using PCR and a Southern blot-based method. The nature of these two mutations and a third type of mutation (e.g., Family B) is further documented below.

### Complex Rearrangements of *MARS2* in ARSAL Patients

To better define the rearrangements, we performed a series of experiments to identify *MARS2* copy number variations (CNVs). In order to circumvent the problem of low average SNP densities in the standard Illumina and NimbleGen CGHs, we designed a custom 845 Kb NimbleGen aCGH array encompassing *MARS2* with an average probe density of 60 nucleotides (nt) to uncover small rearrangements. This high-resolution aCGH was performed on six cases from four families. Note that the *MARS2* gene is surrounded by repetitive DNA, specifically Line 1 and Line 2 elements, but also AT- and TTTA-rich segments, as well as [CGGGG] repeats (Figure 7A).

Based on haplotype analysis (Figure S4), at least three duplication events have occurred in our ARSAL cohort (Figure 7B–C). Indeed, evidence of *MARS2* duplications was uncovered in all six cases that were tested by aCGH (Figure 6D,E,I). The CGH data analysis established that the 268 bp deletion, described above as the c.681Δ268bpfs236X mutation, is part of a duplication since most oligonucleotide probes covering the entire coding sequence of *MARS2* have a log_2_ value (Cy5/Cy3) of ∼0.5–1.0 (Figure 6D–E), whereas compound heterozygous patients should have values of ∼0.2–0.5. To determine whether these complex mutations were segregating in all families and were present in other ARSAL patients, we used seven pre-designed ABI-based Copy Number Assays. Four were located in the *MARS2* coding region and one in each of the nearby genes *PLCL1*, *HSP60*, and *COQ10* (Table S4). *PLCL1*, *HSP60*, and *COQ10* do not exhibit CNVs, whereas *MARS2* duplications were uncovered in all 54 ARSAL cases belonging to 38 families and were not found in 384 control chromosomes (Table S5). Similarly, a Brazilian patient with an ARSAL phenotype also carried a duplication (patient 57 in Table S5).

**Figure 8:**
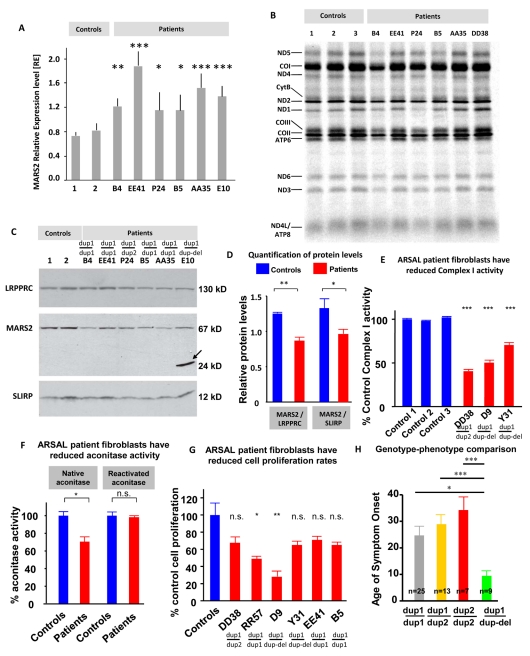
MARS2 mRNA expression, protein levels, mitochondrial protein translation, Complex I, aconitase activity, and cell proliferation. (A) Quantification of MARS2 mRNA expression levels was performed on six ARSAL cases and two control lymphoblast cell lines. Relative expression levels were normalized to GAPDH levels. ARSAL patients expressed up to 3× higher MARS2 mRNA levels compared to controls. (B) Mitochondrial protein synthesis was measured in lymphoblasts and fibroblasts from three controls and six ARSAL patients by pulse-labeling mitochondrial translation products with ^35^S-methionine for 1 h in the presence of emetine, followed by electrophoresis on a 15%–20% linear-gradient polyacrylamide gel. The 13 mitochondrial products are identified at the left of the figure. A generalized mitochondrial translation deficiency is observed in three of the six ARSAL patients tested. ANOVA analysis revealed significance for three of the patient's mitochondrial translation levels: Ctrl 1-B4: **, Ctrl 1-B5: n.s., Ctrl 1-P24: n.s., Ctrl 2-B4: ***, Ctrl 2-B5: n.s., Ctrl 2-P24: *, Ctrl 3-B4: ***, Ctrl 3-B5: *, Ctrl 3-P24: ***. (C) Immunoblotting analysis was performed with antibodies against the proteins indicated at the left of the panel. MARS2 was visualized using a polyclonal antibody. For case E10 carrying the heterozygous deletion (c.681Δ268bpfx236X), the truncated product is detected at the estimated size of 24 kDa (arrow); ARSAL patients (B4, EE41, P24, B5, AA35, and E10) show decreased levels of MARS2 protein at the estimated normal size of MARS2 (67 kDa). The 130 kDa LRPPRC and the 12 kDa SLIRP were used as loading controls. (D) Each patient's MARS2 protein-level intensity from the Western Blot shown in (C) was quantified using ImageJ and divided by the protein-level intensities of LRRPRC and SLIRP. The results were then graphed for the controls and the patients, respectively. (E) Respiratory chain activity for Complex I was measured from patient fibroblast-derived disrupted mitochondria. Mutant mitochondria exhibit deficiency of complex I. Data are expressed as percentage control activity (mean ± s.e.m.). (F) Quantification of native and reactivated aconitase activity for ARSAL patient and control immortalized fibroblasts. Three controls and 6 ARSAL patients were used for the analysis. (G) Quantification of the proliferation rate for the same above-mentioned fibroblasts. (H) Graph showing the average age of onset for the three different genotypes involved.

We hypothesized that the duplications may affect *MARS2* expression levels. Indeed, Northern blots show the expected mRNA size in all patients (Figure S5A), but qPCR quantification assays revealed an increase in mRNA expression in two compound heterozygous and four homozygous ARSAL patients that carry the common duplication (Figure 8A). In addition to the normal *MARS2* mRNA band, we detected small mRNA fragments (∼500 bp) in ARSAL cases but not in the controls (Figure S5B). These bands are suggestive of mRNA instability or aberrant *MARS2* mRNA products. Interestingly, PCR primer walking produced different amplicon lengths that are suggestive of microdeletions ranging from 1 bp to 33 bp in the 250 bp GC-rich 5′ region and interspersed L1-type repetitive elements (Figure 7A). The numerous L1 and L2 elements suggest that the duplications were generated by Fork Stalling and Template Switching (FoSTeS) [48]. However, due to the repetitive nature of the DNA, we were unable to determine precisely where and in which orientation the *MARS2* duplications were located.

In summary, our mapping and CNV data convincingly show that the CNVs are responsible for the ARSAL mutations since none of the 384 non-affected individuals show a CNV in the *MARS2* locus. In addition, the *MARS2* rearrangements do not affect the expression of surrounding genes such as *HSPD1* and *PLCL1* as assessed by aCGH and quantitative PCR (unpublished data). Further evidence of the rare nature of these mutations is the fact that no CNV events have been catalogued for the *MARS2* region in the Database of Genomic Variants (DGV) track. Interestingly, a single Yoruba sequence clone from the Human Genome Structural Variation Project Discordant Clone End track was reported to be discordant from the reference sequence [49]. The discordant clone consists of a 726 bp sequence containing a 276 bp L2 element that mapped within the *MARS2* coding sequence (Figure 7A) and shares the junction breakpoint seen in the ARSAL rearrangements. The CNVs, the quantitative Southern blots, and the Northerns indicate that the rearrangements alter both the dosage of the *MARS2* gene and mRNA. Our CNV results and the presence of numerous local repetitive elements support the hypothesis that regional genomic instability has caused template switching during DNA replication (FoSTeS) (modeled in Figure 7B–C) [48],[50] as well as recombination errors [48],[51],[52].

### MARS2 Protein Levels Are Decreased in ARSAL Patients

To explore the impact of the mutations on protein levels, control and ARSAL patient protein extracts were analyzed by immunoblotting with a mouse polyclonal antibody against the N-terminal end of human MARS2. Despite increased levels of aberrant mRNA transcripts, we find a reduced level of MARS2 protein in all tested patients, ranging from 40%–80% of normal, using mitochondrial proteins encoded in the nucleus as loading controls (Figure 8C, quantified in 8D). Importantly, carriers of the deletion (but none of the other patients or controls) produce the expected 24 kDa truncated protein in addition to the normal band (black arrow in Figure 8C, Figure 7C). The level of the truncated MARS2 protein is at least three times higher than the level of the wild-type protein found in controls. The Western blot data combined with Northern blot data argue that some *MARS2* transcripts are not translated, possibly because of a post-transcriptional regulatory event such as an RNA-mediated interference of translation (Figure 7B).

### Mitochondrial Translation and Respiration Are Decreased in ARSAL Patients

To test whether mutations in *MARS2* affect mitochondrial translation, we pulse-labeled the mtDNA-encoded polypeptides in patient and control immortalized lymphoblast lines as previously reported (Figures 8B, Table S6) [53],[54]. Of the six patients tested, three showed a translation deficiency. These three patients are homozygous for the common mutation (*Dup1/Dup1*) (cases B4, B5, and P24) and correspond to the most severe cases diagnosed at the ages of 6, 3, and 9, respectively (Table S5). Two patients with control levels of translation were compound heterozygotes for two different duplications (EE41, AA35). These patients were clearly less severely affected and were diagnosed as adults at the ages of 36 and 26, respectively. In addition, other clinical variables such as loss of walking ability (Table S5) correlate with the extent of the translation defect in lymphoblasts. Despite the relative decrease of MARS2 levels, no effect on the steady-state levels of mitochondrial tRNA^methionine^ was uncovered (Figure S6A–B), suggesting that the amino-acylation defect does not destabilize the cognate tRNA.

To address if and how knockdown of *MARS2* in cells affects translation of mitochondrial proteins, we reduced the levels of MARS2 in HEK293 cells with three different shRNAs (Figure S6C). A severe knockdown (SH-452) clearly affects mitochondrial protein translation (Figure S6E), whereas a less severe reduction in MARS2 (SH-152) does not cause an obvious reduction in mitochondrial protein translation when compared to wild-type controls. Similarly, overexpression of MARS2 had no effect on mitochondrial protein translation (Figure S6D,F). Hence, unless the MARS2 protein level is reduced beyond a certain level, levels of mitochondrial translation are not obviously affected. We did not identify a significant difference in MARS2 protein levels between the patients of different genotypes, although most patients with the *Dup1/Dup1* genotype have slightly lower MARS2 levels than the other patients (unpublished data). Finally, consistent with our findings in *Aats-met* mutant flies, cultured patient fibroblasts displayed reduced Complex I activity, increased ROS levels, and concomitantly decreased cell proliferation rates (Figure 8E–G).

Finally, we performed an examination of the genotype-phenotype relationship using the age of symptom onset as a measure of the severity of the disease and noted that patients carrying the duplication-deletion tend to have an earlier onset (Figure 8H).

## Discussion

Although mitochondria play an important role in all cells, neurons and muscles are typically more affected by mitochondrial dysfunction. The isolation of *Aats-met* mutations in a mosaic FLP-FRT eye screen on 3R resulting in PR degeneration as well as other mutations that cause neurodegenerative phenotypes on the X-chromosome (Shinya Yamamoto et al., personal communication) attest to the power of these screens. Many of these genes encode mutations in mitochondrial genes (unpublished data). Hence, this strategy may allow us to uncover other human neurodegenerative diseases and allow us to probe more systematically into the biology of other human disease genes.

Based upon the endosymbiont theory, most of the mitochondrial genome translocated to the nucleus, leaving only 13 coding genes and a set of ribosomal and transfer RNAs in the mitochondrial genome [55],[56]. Among the genes that translocated to the nucleus was a family of mitochondrial tRNA synthetases, including the mitochondrial methionyl-tRNA synthetase—*Aats-met* in *Drosophila* and *MARS2* in humans. Interestingly, there is a growing list of mitochondrial-targeted proteins that when mutated cause neurological diseases [57]. Besides *MARS2*, mutations in other mitochondrial tRNA synthetases are being linked to heterogeneous human diseases (Table S7), highlighting their importance for human health [58],[59],[60],[61],[62],[63],[64].

Severe allelic combinations of *Aats-met* in flies cause lethality, while partial loss of function alleles exhibit phenotypes that can be much more easily related to ARSAL. These partial loss-of-function mutations will also allow us to perform modifier screens to identify associated genes as well as drugs. Indeed, our preliminary efforts to treat these flies with antioxidants indicate that such screens are feasible.

### Mutations in *MARS2* in ARSAL Patients

ARSAL exhibits clear inter- and intrafamilial variability reminiscent of Friedreich Ataxia [28],[65],[66]. In the present study, we report a group of 54 affected French-Canadian cases belonging to 38 families with a mean age of onset of 24.4 (2–59) in which we uncovered complex genomic *MARS2* rearrangements (Table S5, Figure 7B–C). The mutations are complex genomic *MARS2* rearrangements that always include a gene duplication event. Duplications were found with similar breakpoints located in a GC-rich 5′ UTR sequence and in a 3′ non-coding region. The junctions created by the rearrangements are located outside the coding region of *MARS2* or other known genes and do not disturb the expression of neighboring genes as demonstrated by CNV assays and quantitative PCR. The 3′ UTR of *MARS2* also seems affected by putative disruptions of regulatory elements at the breakpoint junction (Figure 7A). This duplication was neither detected in 384 controls, nor described in the structural variation database. Moreover, in all families for which we have affected and unaffected relatives available for genetic analysis, the presence of the rearrangement (CNV) segregated with the disease. These data strongly argue that mutations in *MARS2* are the cause of ARSAL, and this in turn is supported by an increase in message levels of *MARS2* mRNA, reduced levels of MARS2 protein, and a reduction in mitochondrially translated proteins and Complex I activity in patients.

The high prevalence of repetitive sequences at both breakpoint junctions, including many long-interspersed elements (LINES) at the 5′ region of *MARS2*, and several AT-rich repeat sequences are likely to have mediated the rearrangements (Figure 7A) [67],[68]. Despite the increased mRNA levels, we observed decreased MARS2 protein levels. The increased mRNA levels may be due to the duplications of the gene as well as duplications of regulatory elements in the CpG island at the 5′ end of the *MARS2* gene. Consistent with recent studies, analysis of the *MARS2* genomic structure reveals a functional CpG island (Figure 7A) [69],[70]. CpG islands act as constitutive promoters of housekeeping genes and are methylated to silence transcription [71]. These findings suggest that the *MARS2* duplications may dysregulate transcription, possibly by affecting the size, composition, or methylation ability of these islands.

The decrease in protein levels contrasts with the increase in message. The simplest hypothesis is that the gene duplications were caused by FoSTeS, and a small fragment of DNA encoding some of the 5′ or 3′ UTRs was inverted. This inverted segment may affect mRNA stability and/or translation of MARS2 via an RNAi-mediated mechanism. Indeed, FoSTeS has been shown to result in duplicated inverted segments [72],[73]. Unfortunately, the highly repetitive nature of the DNA surrounding the *MARS2* gene did not allow us to document this inversion.

### Mitochondrial Dysfunction/ROS in *Aats-met* Mutants and ARSAL Patients

Our data suggest that decreased levels of Aats-met/MARS2 protein or protein function lead to a subtle reduction in mitochondrial translation in humans and problems with mitochondrial function in flies and humans. The partial loss of Aats-met protein seems to lead to the accumulation of misfolded proteins in mitochondria, triggering a mitochondrial Unfolded Protein Response (UPR^mt^) (Figures S3A–D, S3G). Mutant flies and patient cells also exhibit abnormal mitochondrial physiology, most notably a rather surprisingly mild reduction in Complex I activity, as well as accumulation of ROS (Figures 5A–C, 8D–E). The reduction in Complex 1 activity is consistent with the observation that 7 of the 13 mitochondrially encoded proteins are incorporated in Complex 1.

The brain tissue of *Aats-met* mutants contains lipid droplets that are almost never observed in wild type neurons and glia. Such an increase in lipid droplets, potentially related to a lipid metabolism defect, was also recently observed in a 12-y-old girl exhibiting progressive muscle degeneration and autoimmune polyendocrinopathy and was determined to have cosegregating mutations in MARS2's cognate tRNA, mitochondrial tRNA^methionine^, as well as COX III [74], as well as in patients with other mitochondrial diseases such as Leigh Syndrome, Alpers Disease, and Lethal Infantile Mitochondrial Disease [75].

*Aats-met/MARS2* mutations do not solely affect neuronal function and survival. Indeed, severe allelic combinations affect cell proliferation, but not cell growth and apoptosis. These data are consistent with the role of increased levels of ROS in the activation of the G1-S checkpoint via the JNK signaling pathway, blocking cell cycle progression [12]. ROS has been shown to play a role in the regulation of the cell cycle, both in its promotion and blockage [44]. Importantly, several of the patient cell lines, similar to what was observed in flies, also exhibit reduced cell proliferation and increased ROS (Figure 8F–G).

The clinical features of ARSAL clearly argue that the neurons, glia, and muscles are more affected than other tissues or organs (Table S5) [28]. Indeed, ARSAL patients exhibit ataxia, severe cerebellar and some cerebral atrophy, dystonia, and leukodystrophy. Flies that carry weak allelic combinations also exhibit a progressive demise of the muscles and brain, as can be seen in Figures 2, 3, and S1. In both patient cells and flies we observe decreased levels of Complex I activity and increased levels of ROS. The ability to partially suppress the morphological defects in flies with various antioxidant compounds is noteworthy. Normally, ROS levels are tightly controlled and known to play important roles in signaling pathways, including the HIF-1α, JNK, NFκB, TNF-α, and NADPH Oxidase pathways [76]. The production of excessive levels of ROS may also play a prominent role in other neurodegenerative diseases [77],[78],[79]. Finally, Vitamin E deficiency as a cause of an ataxia (AVED, OMIM #277460) further supports a role for ROS in hereditary cerebellar diseases [80].

In conclusion, mutations in *Aats-met* in flies or reduced levels of MARS2 protein in humans result in aberrant translation of the Respiratory Chain and concomitant production of ROS. These ROS are especially damaging to neurons, as evidenced by our finding that the ERG progression of the *Aats-met* mutants can partially be suppressed by antioxidants (unpublished data). This ROS also has the effect of reducing cell proliferation, a phenotype that can also be suppressed by antioxidants (Figure 5D–E). Our model is summarized in Figure S7. It remains to be determined if antioxidants will prove beneficial for ARSAL patients.

## Materials and Methods

### Clinical Information and Ethics Statement

All probands and family members underwent a detailed neurological examination by experienced neurologists. All medical records and imaging were reviewed. All families were of French-Canadian ancestry except for one Brazilian family. None of the families were known to be consanguinous. All MRIs were reviewed by J.L. This project was approved by the Institutional Ethics Committee of CRCHUM. Informed consent was obtained from all patients, all family members, and controls. Genomic DNA was extracted from blood or saliva using standard procedures (Oragene, DNA Genotek).

### Strains

Mutagenesis of chromosome 3R was performed as described previously [26]. The genotypes of *FB* and *HV* are: *y w; FRT82B Aats-met^FB^/TM3* and *y w; FRT82B Aats-met^HV^/TM3*. *P*-element/deficiency mapping was performed as described [30]. The genotype of the *Df* stock is: *y w*; *Df(3R)Exel7321/TM3, hs-hid* [81]. The genotype of the *piggyBac* is: *y w*; *FRT82B pBac^c00449^/TM3, hs-hid* [31]. The control strain used was *y w*; *FRT82B isogenized*. To generate mutant eye clones, *y w eyFLP*; *FRT82B w^+^ cl/TM3* was crossed to *y w*; *FRT82B Aats-met^FB^/TM3* and *y w*; *FRT82B Aats-met^HV^/TM3*. Transheterozygous escapers were generated in large numbers by raising the larvae/pupae at 18°C. They were subsequently raised at room temperature and transferred and scored every 2–3 d for aging experiments. Heat-shock clones were generated using *y w hsFLP*; *FRT82B ubi-GFP^nls^/TM6B*. For rescue experiments, *y w*; *Act5C-Gal4/CyO* was used. The UAS-p35 stock used to inhibit apoptosis has been described [82]. Climbing assays were performed exactly as described [83]. Unless indicated, stocks were obtained from the Bloomington *Drosophila* Stock Center (BDSC) and are listed on FlyBase (http://flybase.bio.indiana.edu).

### Drug Studies

AD4 (N-acetylcysteine amide) and Vitamin E (MP Biomedicals) were dissolved in standard fly food. The same food batch without drug supplementation was used for the control.

### Electrophysiology

ERGs were recorded as described previously [26].

### Microscopy

Images of eyes and pupae were taken with a MicroFire camera (Optronics) mounted on a Leica MZ16 microscope. TEM of photoreceptors was performed as described previously [24]. At least five animals were analyzed. Thick sections were prepared for inspection of sample integrity. For quantification, 18–20 photoreceptor cartridges for each genotype were analyzed. Thick sections of the optic lobe (Figure S1) were visualized using a microscope (Imager.Z1; Carl Zeiss, Inc.), camera (AxioCam MRm; Carl Zeiss, Inc.), AxioVision release 4.3 software (Carl Zeiss, Inc.), and the Plan-Apochromat 20× NA 0.75 lens.

### Molecular Biology

For sequencing, DNA from mutant larvae was sequenced (Macrogen) and analyzed (DNAStar). The *Aats-met* cDNA (DGC clone GH13807) and the human *MARS2* cDNA (Open Biosystems MHS4426-99239542) using iProof polymerase (Bio-Rad) and appropriate oligos (with a Kozak sequence) were subcloned into the pUAS-attB vector and injected into embryos containing the VK37 attP site [84].

### Mitochondrial Physiology and Labeling Experiments

Third instar larvae were homogenized in cold mitochondrial isolation buffer using a Dounce homogenizer (Kontes), filtered through cheesecloth, and centrifuged at 150 G, then 9,000 G. Oxygen consumption of mitochondria was measured (Clark microelectrode (YSI Life Sciences)), recorded (PowerLab data recorder), and analyzed (ADInstruments LabChart). Rates (ng atomic oxygen/min/mg mitochondrial protein) were expressed as percentage control activity. Polarography was performed for six independent mitochondrial isolations. For enzymology, 3^rd^ instar larval mitochondria were sonicated as above. Spectrophotometric kinetic assays were performed (monochromator microplate reader (Tecan M200)). Complex I activity was determined by measuring NADH oxidation (340 nm), Complex II activity by measuring DCIP reduction (600 nm), Complex III activity by measuring CytC reduction (550 nm), Complex IV activity by measuring CytC oxidation (550 nm), and Citrate synthase activity by measuring DTNB reduction (412 nm) coupled to acetyl-CoA reduction. All activities were calculated as nmoles/min/mg protein and expressed as percentage control. Six independent samples for each genotype were tested. The activity of mitochondrial aconitase was measured on the basis of conversion of citrate into α-ketoglutarate coupled with NADP reduction (Sigma) and was normalized for total protein [45]. Activity was measured in the native state and after “reactivation” by incubating mitochondria in ferrous ammonium sulfate for 5 min before performing the assay. In vitro labeling of mitochondrial translation products was performed as described previously [53].

### Immunohistochemistry

Immunohistochemistry was performed as previously described [85]. Anti-BiP (1∶200) [41], anti-*Drosophila* Hsp60 (1∶200) [39], anti-Dlg (1∶50) [86], anti-cleaved caspase 3 (1∶500) (Cell Signaling), anti-PhosphoHistone 3 (ab5176) (1∶1,000) (Abcam), anti-Fasciclin II (1D4) (1∶10) [87], anti-Elav (1∶500) (7E8A10) [88], anti-Brp (Nc82) (1∶100) [89], and anti-Repo (8D12) (1∶10) [90] were used. Secondary antibodies conjugated to Cy3, Cy5, or Alexa 488 (Jackson ImmunoResearch and Invitrogen) were used at 1∶250. For anti-PH3 quantification, homozygous *FB* clones were stained with anti-PH3 to mark cells undergoing DNA synthesis. The largest box possible was made of the disc, and PH3-positive cells were documented with red dots in heterozygous tissue or purple dots in the homozygous tissue. The area was then determined for both, and paired Student *t* tests were performed for each of five discs to compare the difference in the number of PH3-positive cells in homozygous tissue versus heterozygous tissue. A total of 20 pairs of clones and their twin spots for each genotype+temperature were measured.

### SNP Genome Scan, CNV, and CGH Arrays

A SNP genome-wide scan with the Illumina HAP300 SNP chip was conducted at the Genome Quebec Innovation Center, McGill University (Montreal, Canada) on nine affected individuals and six non-affected family members. BeadStudio Software was used as an analysis tool for genotyping, homozygosity, and loss of heterozygosity analysis. Copy number analysis was performed using the PennCNV program.

We used seven pre-designed ABI-based Copy Number Assays for human CNV screening; four were located in the *MARS2* coding region, one in each coding sequence of the surrounding genes (*PLCL1*, *HSPD1*, and *COQ10*) (Table S4). Each reaction was performed in quadruplicate on a 384-well PCR plate with the ABI Copy Number Reference Assay (*RNaseP*). CopyCaller (Applied Biosystems) was used for data analysis, and all steps were done according to instructions. NimbleGen CGH-array was performed using a chr2 specific fine-tilling oligonucleotide (HG18 CHR2 FT) to detect chromosomal changes. The median probe spacing was ∼500 bp. Custom high-resolution NimbleGen's 12×135K CGH arrays (38,725 probes per array on Chr2) were designed to cover the entire 0.845 Mb surrounding *MARS2* [91],[92]. The median probe spacing was 1 bp.

### Mutation Analysis and qRT-PCR

Primers were designed (Table S4) using Primer 3 or ExonPrimer (see URLs section below). Sequences were analyzed on an ABI3730 Genetic Analyzer (Applied Biosystems). RNAs were treated with DNase I to avoid genomic DNA amplification. Reverse transcription was performed using 3 µg total RNA using random hexamers, OligodT, and Superscript III (Invitrogen) according to the vendor's protocol. We prepared cDNAs from total RNA and performed cDNA analysis by PCR with the primers as indicated in the manufacturer's protocol. Purified PCR fragments were subcloned into pCR II-TOPO TA cloning kit (Invitrogen) (Table S4).

Quantitative real-time PCR experiments were performed using an ABI PRISM 7900 HT (Applied Biosystems) on genomic DNA and cDNAs. Transcript-specific primers were designed with Primer Express software (Applied Biosystems). The PCR conditions and analysis of the obtained data were optimized using published protocols [93],[94]. The cycle of threshold value (Ct) was normalized to the transcripts for the housekeeping genes *β-globulin* and *GAPDH*. We performed calculations as described previously [93],[94]. Primer sequences are shown in Table S4 [95].

### Cell Culture and RNA Extraction

Cell lines were maintained under normal condition (37°C, 5% CO_2_) in standard culture media (DMEM containing 10% FBS and 100 µg/ml Pen-Strep and 50 µg/ml gentamicine). RNA extraction was performed using TRIZOL (Invitrogen). To measure the fibroblast cell proliferation rate, fibroblasts from the three control and seven patient cell lines were cultured in 12 well plates as described earlier. They were plated at the same pre-determined concentration (900 cells/ml) using a hemocytometer as a guide and were counted using a Beckman Coulter Vi-Cell XR2.03 cell viability analyzer after 48 h and then quantified.

### Immunoblotting

An N-terminal mouse polyclonal antibody was obtained from Abnova (MARS2-H00092935-Q01) and used at 1∶1,000. We used antibodies against LRPPRC and SLIRP as loading controls. The LRPPRC polyclonal antibody was prepared by Zymed Laboratories (#295–313) and used at 1∶3,000. The polyclonal antibody against SLIRP was used at 1∶1,000 (Abcam #ab51523). Protein was extracted from cultured cells, and 20 µg were subjected to SDS-PAGE and transferred to nitrocellulose membranes (Millipore). The blot was probed overnight at 4°C with the primary antibodies and then probed for 1 h at room temperature with anti-rabbit IgG-HRP secondary antibody (1∶10,000; Santa Cruz Biotechnology). We visualized proteins using ECL Western Blot detection reagent (PerkinElmer).

### Northern and Southern blotting Analysis

10 µg of total RNA extracted from control and patient lymphoblasts were run on a 10% polyacrylamide gel containing 7 M urea, followed by transfer to Hybond N+ membrane (GE Healthcare). Pre-hybridization and hybridization were carried out in EXPRESS-Hyb solution (Clontech) according to the manufacturer's instructions. The oligonucleotides used for the generation of the ^32^P-labeled probes had the following sequences: 5′-TGGTAGTACGGGAAGGGTATAACC-3′ for tRNA-Met and 5′-TGGTATTCTCGCACGGACTACAAC-3′ for tRNA-Glu. The commercial cDNA of *MARS2* was digested by *Xho1*/*Pst1* (OriGene; SC100504) and oligonucleotides of complement and reversed *MARS2* sequences.

Southern blot analysis was performed to assess *MARS2* genomic rearrangements. Southern blots were produced using standard protocol with control and mutation carrier DNA. The following restriction enzymes for DNA digestion were used: *AflIII*, *ApaI*, *BamHI*, *BglII*, *HindIII*, *KpnI*, *NcoI*, *PstI*, and *XhoI*. A cDNA probe was obtained from commercial human cDNA digested with *XhoI*/*PstI* (OriGene; SC100504). The blots were hybridized with a ^32^P-labeled *MARS2* cDNA probe as described (http://www.protocol-online.org/cgi-bin/prot/view_cache.cgi?ID=2746).

### Statistical Analysis

Statistical analysis was performed using Excel (Microsoft) and Prism (GraphPad). Except where otherwise mentioned, unpaired two-tailed Student *t* tests were used. Percentage protein similarity was determined using BlastP (NCBI).

### Accession Numbers

We used sequences for *MARS2* with accession numbers NM_138395.2 and NP_612404.1.

## Supporting Information

Figure S1 ***FB*** **and** ***HV*** **mutants correspond to** ***Aats-met*** **and degenerative phenotypes in** ***HV/FB*** **escapers.** (A) After rough mapping with seven widely spaced *P*-elements were used (unpublished data), four *P*-elements were used to refine the locus. Deficiency complementation tests in this area were performed to identify four overlapping ones that uncovered a 120 Kb region. (B) Available lethals were ordered and crossed, with a *PiggyBac* insertion in the *Aats-met* gene failing to complement. (C) Light micrograph of a resin-embedded thick section of a 3-wk-old *HV/FB* escaper fly's optic lobe, showing vacuolization (arrows) and retinal degeneration (arrowhead). (D) Light micrograph of a resin-embedded thick section of a 3-wk-old *Act-Gal4/UAS-Aats-met*; *FB/Df* rescued fly's optic lobe, showing normal features. (E) Light micrograph of a resin-embedded thick section of a control (FRT82B iso) retina (100×) stained with toluidine blue to mark the lipids. (F) A micrograph of a mutant (FB) retina stained with toluidine blue, showing large lipid droplets in the glia (indicated by red arrows).
(TIF)

Figure S2 **Cell proliferation is impaired, but apoptosis is not affected in mutant clones.** (A) An example of one of the five wing imaginal discs quantified for cell proliferation, as described in the Materials and Methods section. (B) A representative homozygous mutant clone in the wing disc marked negatively with GFP is shown. (C) Yellow dashed lines denote the position of the mutant clone from B and is stained with αCleaved Caspase 3, showing that there is no increase in Caspase 3 levels in the clones. (D–F) Larval brains of late third-instar control, FB/Def, and actin>P35; FB/Def larvae are shown, indicating that both mutant and apoptosis-inhibited mutant larval brains are both similarly small. (G) Quantification of the above is graphically displayed. Scale bars are 50 µm for (A) and (D–F) and 5 µm for (B–C).
(TIF)

Figure S3 **Upregulation of the mitochondrial unfolded protein response without concomitant cytoplasmic UPR response.** (A) A control adult eye (*y w eyFLP*; *FRT82B iso/FRT82B w^+^ cl*) stained with anti-Hsp60, a protein that has been implicated as a marker of the UPR^mt^. (B) A mutant eye (*y w eyFLP*; *FRT82B Aats-met^FB^/FRT82B w^+^ cl*) stained with anti-Hsp60 shows a marked increase in staining in the retina and lamina (where the flippase is expressed). The dashed white lines mark the lamina, and the green lines mark the retina. (C–D) Heat-shock clones of *Aats-met^FB^* were generated in the wing imaginal disc (negatively marked for GFP) and stained for anti-Hsp60 (red), showing elevated levels of Hsp60 in mutant clones. Genotype: *y w hsFLP*; *FRT82B Aats-met^FB^/FRT82B Ubi-GFP^NLS^*. (E–F) Similar experiments were done with anti-BiP, a marker of the cytoplasmic UPR, showing unchanged levels in *Aats-met^FB^* mutant clones. (G) Quantification of the increased levels of Hsp60 in mutant clones versus neighboring tissue. (H) Quantification of the eye surface area of eyes carrying *HV* and *FB mutant* clones, untreated and treated with 20 µg Vitamin E, showing that it suppresses the small eye phenotype.
(TIF)

Figure S4 **Homozygosity mapping by SNP microarray analysis.** Homozygosity and haplotype analysis of DNA samples from nine patients belonging to five ARSAL families. Homozygosity spans over 50 Mb in Family B (unpublished data). Three common haplotypes on chromosome 2q33–34 surrounding the *MARS2* region were identified (indicated in light grey for Dup1, dark grey for Dup2, and blue for the Dup-Del). An overlapping region for the three haplotypes was identified (black bar).
(TIF)

Figure S5 **Northern blots of ARSAL patients.** (A) Northern blot of six patients' and three controls' lymphoblasts is displayed. mRNAs of the same size (arrow) were detected by using a cDNA probe covering the entire *MARS2* coding sequence for all cases examined. (B) Northern blot of patients' and controls' lymphoblasts is displayed. mRNA degradation (arrowheads) was detected using a cDNA probe covering 875 bp *MARS2* coding sequence for all patients examined but not in the controls. Red lettering indicates patients and blue lettering refers to controls.
(TIF)

Figure S6 **Mitochondrial tRNAs are stable in ARSAL patient cells, and loss of MARS2 in cells results in impaired mitochondrial translation.** (A) Total steady-state levels of mitochondrial (mt) tRNA-met in patients and controls are similar, suggesting that decreased amino-acylation does not interfere with the stability of mt tRNA-met. mt tRNA-glu was used as a loading control. (B) Quantification of the mitochondrial methionyl-tRNA level relative to mitochondrial glutamic acid-tRNA is shown. MARS2 protein levels and mitochondrial protein translation. (C) Western blot of MARS2 protein performed on HEK293 cells transfected with shRNA constructs against human MARS2. Relative expression levels were normalized to prohibitin levels and the two controls (Mock, Alexa). shRNA constructs reduce MARS2 protein levels (SH-451: 15% of control, SH-452: 25%, SH-152: 75%). (D) Western blot of MARS2 protein in HEK293 cells expressing a MARS2-GFP transgene that results in 2× normal expression. (E) Mitochondrial protein synthesis was measured in siRNA experiments by pulse-labeling mitochondrial translation products with ^35^S-methionine for 1 h in the presence of emetine, followed by electrophoresis on a 15%–20% linear-gradient polyacrylamide gel. The 13 mitochondrial products are evident. A significant generalized mitochondrial translation deficiency is observed when the protein level of MARS2 is reduced to 25% of controls. There was too much cell death caused by SH-451 expression to perform the translation assay. (F) Mitochondrial protein synthesis was measured after GFP-MARS2 overexpression in HEK293 cells by pulse-labeling mitochondrial translation products with ^35^S-methionine for 1 h in the presence of emetine, followed by electrophoresis. The experiment was conducted 3 times. No impact on mitochondrial translation is observed.
(TIF)

Figure S7 **Pathologic** ***Aats-met*** **model.** The model can be summarized as follows. Mutations in Aats-met result in impaired translation of the 13 components of Complexes I, III, IV, and V that are encoded in the mitochondrial genome. This results in impaired complex formation, a mitochondrial UPR, and an uncoupled respiratory chain. The resulting ROS causes tissues to degenerate, most notably neurons and muscle, and also affects cell proliferation via its effect on the cell cycle (JNK signaling). These effects of degeneration and cell proliferation can be partially suppressed by antioxidant supplementation.
(TIF)

Table S1 **Respiration rates of isolated mitochondria from control and** ***Aats-met*** **mutant larvae.** The respiration rates for isolated mitochondria from 3^rd^ instar larvae are listed for each of the genotypes used—control (*FRT82B isogenized*), *HV*/*Df*, and *FB/Df*, with means and standard deviations listed.
(PDF)

Table S2 **Respiratory chain enzyme activities of isolated sonicated mitochondria from control and** ***Aats-met*** **mutant larvae.** The enzyme activities, with means and standard deviations, for Complexes I, II, III, IV, and Citrate Synthase are listed. The genotypes used were control (*FRT82B isogenized*), *HV*/*Df*, and *FB/Df*.
(PDF)

Table S3 ***Drosophila*** **and human mitochondrially encoded proteins possess many methionines.** The *Drosophila* and human mitochondrially encoded proteins are listed in the first column. The Respiratory Complex that they each belong to is listed in the second column. The number of methionines and methionine percentage of the *Drosophila* proteins is listed in the third column. The number of methionines and methionine percentage of the human proteins is listed in the fourth column.
(PDF)

Table S4 **Primers used.** The primers used for quantitative PCR, sequencing of the MARS2 genomic region and cDNA, and for the CNV assays are displayed.
(PDF)

Table S5 **ARSAL patients' genetic variations and clinical characteristics.** ARSAL patients are listed. Alongside them are their family identifiers, gender, their genetic variations, the method by which their mutations were identified, the age of symptom onset, and the presence or absence of 11 clinical/imaging characteristics.
(PDF)

Table S6 **Mitochondrial protein synthesis.** Quantification of mitochondrial protein synthesis shows a generalized deficiency in the patients homozygous for the common mutation (54%, 67%, and 79% of the average of controls). On the other hand, patients who are compound heterozygous for *MARS2* mutations have normal mitochondrial translation (89%, 107%, and 118% of the average of controls).
(PDF)

Table S7 **AARS diseases.** These 12 AARS-related diseases, the responsible genes, and their documented clinical phenotypes are listed. Note that those genes with a “2” at the end of their name (i.e., MARS2, DARS2, RARS2, YARS2, HARS2, AARS2, SARS2, and LARS2) are purely mitochondrial tRNA synthetases. GARS and KARS encode both the mitochondrial and cytoplasmic tRNA synthetases based on the splice forms translated, and YARS and AARS encode purely cytoplasmic synthetases.
(PDF)

## Abbreviations

Aats-met: Aminoacyl-tRNA synthetase-methionine
aCGH: array Comparative genomic hybridization
ARSAL: Autosomal Recessive Spastic Ataxia with Leukoencephalopathy
CNV: copy number variation
Df: Deficiency
EMS: ethyl methanesulfonate
ERG: electroretinogram
ETC: Electron Transport Chain
FoSTeS: Fork Stalling and Template Switching
Kb: kilobase
MARS2: Methionyl Aminoacyl-tRNA Synthetase 2
NMNAT: nicotinamide mononucleotide adenylyltransferase
RCR: respiratory control ratio
SNP: Single Nucleotide Polymorphism
TEM: Transmission Electron Microscopy
UPR: Unfolded Protein Response
