# Supplementary figures and images for: Mutations in the Mitochondrial Methionyl-tRNA Synthetase Cause a Neurodegenerative Phenotype in Flies and a Recessive Ataxia (ARSAL) in Humans
Source: PLoS Biol. 2012 Mar 20;10(3):e1001288. doi: 10.1371/journal.pbio.1001288 (PMC3308940; doi:10.1371/journal.pbio.1001288)

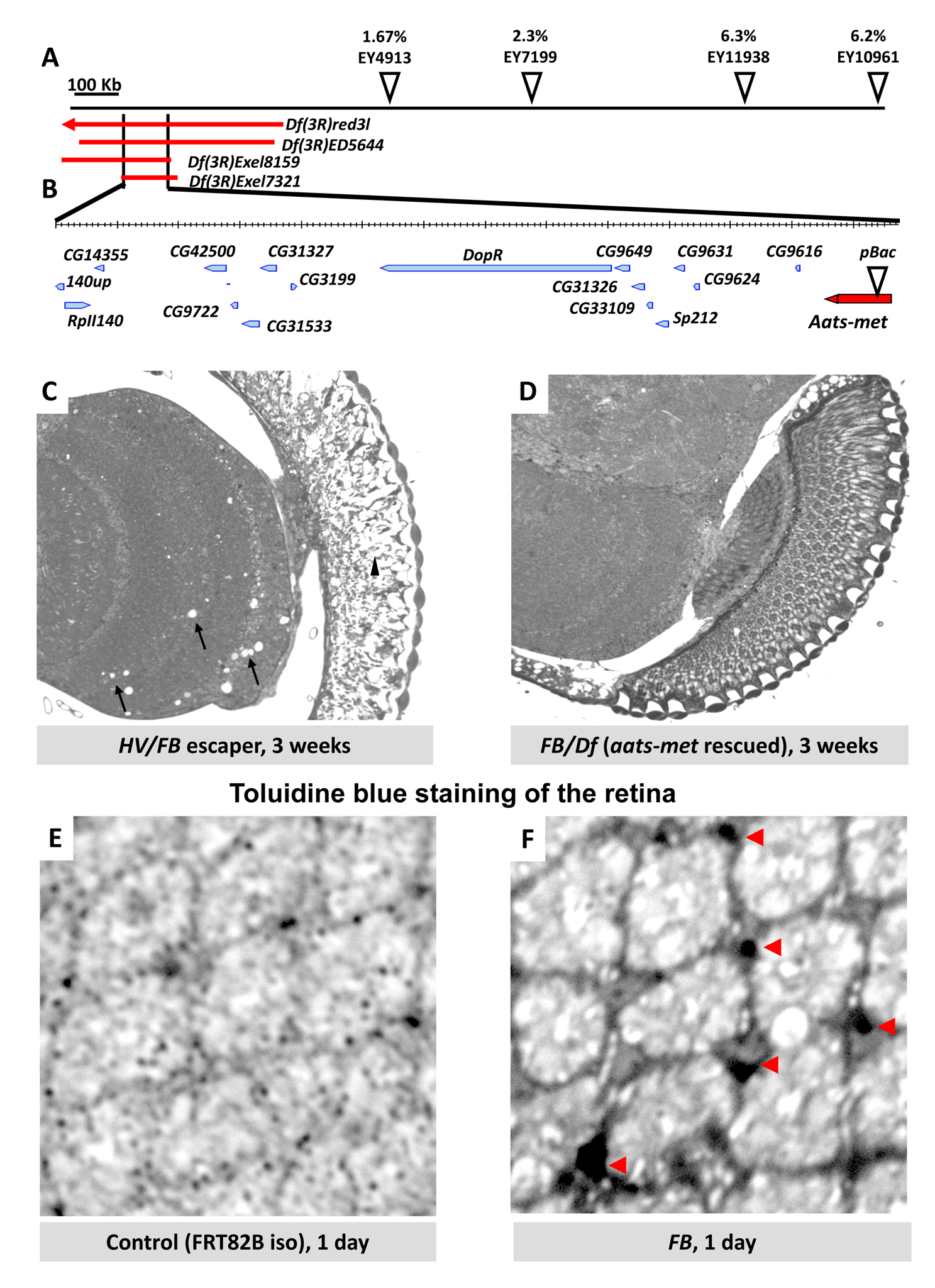

Supplement: Figure S1 — FB and HV mutants correspond to Aats-met and degenerative phenotypes in HV/FB escapers. (A) After rough mapping with seven widely spaced P-elements were used (unpublished data), four P-elements were used to refine the locus. Deficiency complementation tests in this area were performed to identify four overlapping ones that uncovered a 120 Kb region. (B) Available lethals were ordered and crossed, with a PiggyBac insertion in the Aats-met gene failing to complement. (C) Light micrograph of a resin-embedded thick section of a 3-wk-old HV/FB escaper fly's optic lobe, showing vacuolization (arrows) and retinal degeneration (arrowhead). (D) Light micrograph of a resin-embedded thick section of a 3-wk-old Act-Gal4/UAS-Aats-met; FB/Df rescued fly's optic lobe, showing normal features. (E) Light micrograph of a resin-embedded thick section of a control (FRT82B iso) retina (100×) stained with toluidine blue to mark the lipids. (F) A micrograph of a mutant (FB) retina stained with toluidine blue, showing large lipid droplets in the glia (indicated by red arrows). (TIF) [file pbio.1001288.s001.tif]

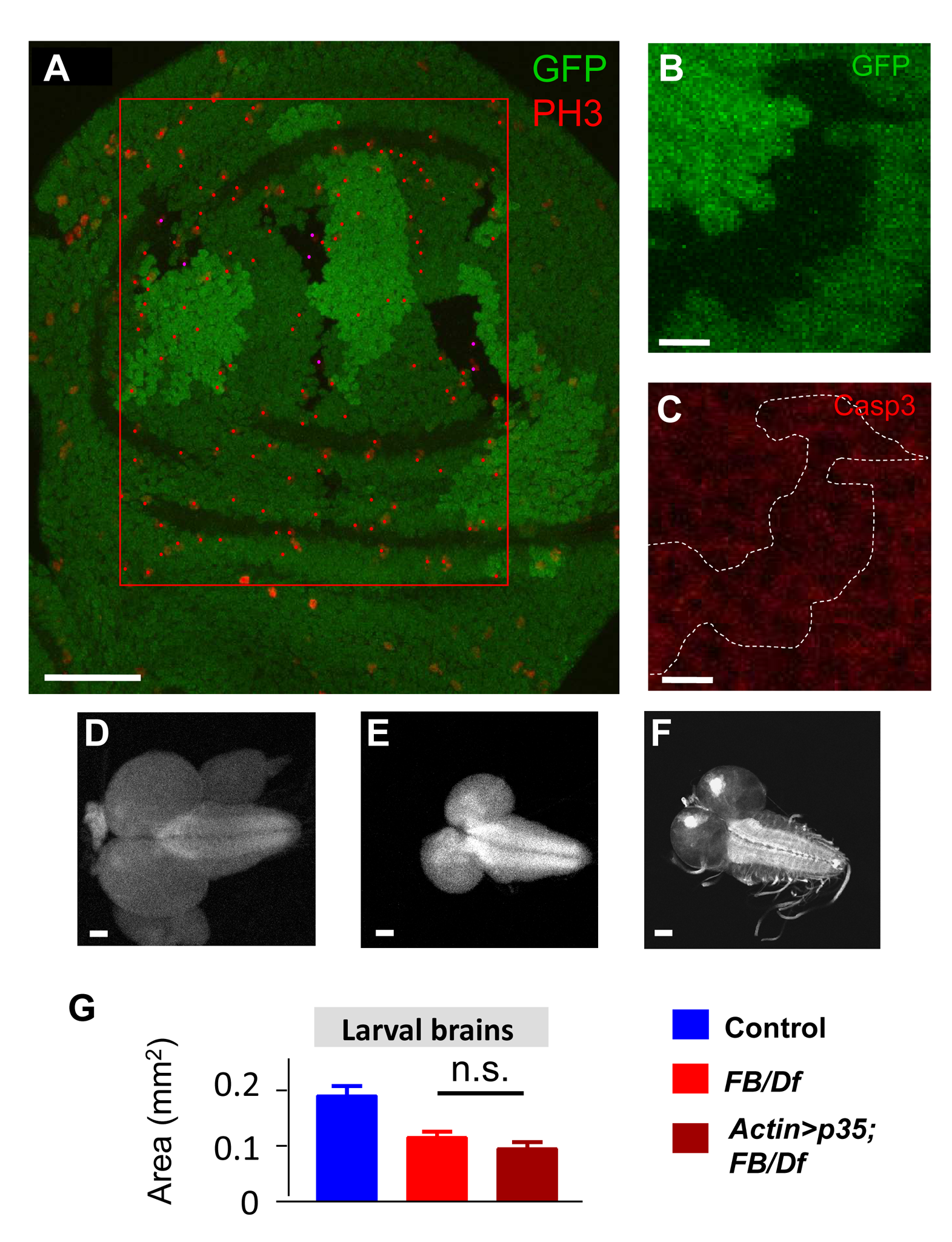

Supplement: Figure S2 — Cell proliferation is impaired, but apoptosis is not affected in mutant clones. (A) An example of one of the five wing imaginal discs quantified for cell proliferation, as described in the Materials and Methods section. (B) A representative homozygous mutant clone in the wing disc marked negatively with GFP is shown. (C) Yellow dashed lines denote the position of the mutant clone from B and is stained with αCleaved Caspase 3, showing that there is no increase in Caspase 3 levels in the clones. (D–F) Larval brains of late third-instar control, FB/Def, and actin>P35; FB/Def larvae are shown, indicating that both mutant and apoptosis-inhibited mutant larval brains are both similarly small. (G) Quantification of the above is graphically displayed. Scale bars are 50 µm for (A) and (D–F) and 5 µm for (B–C). (TIF) [file pbio.1001288.s002.tif]

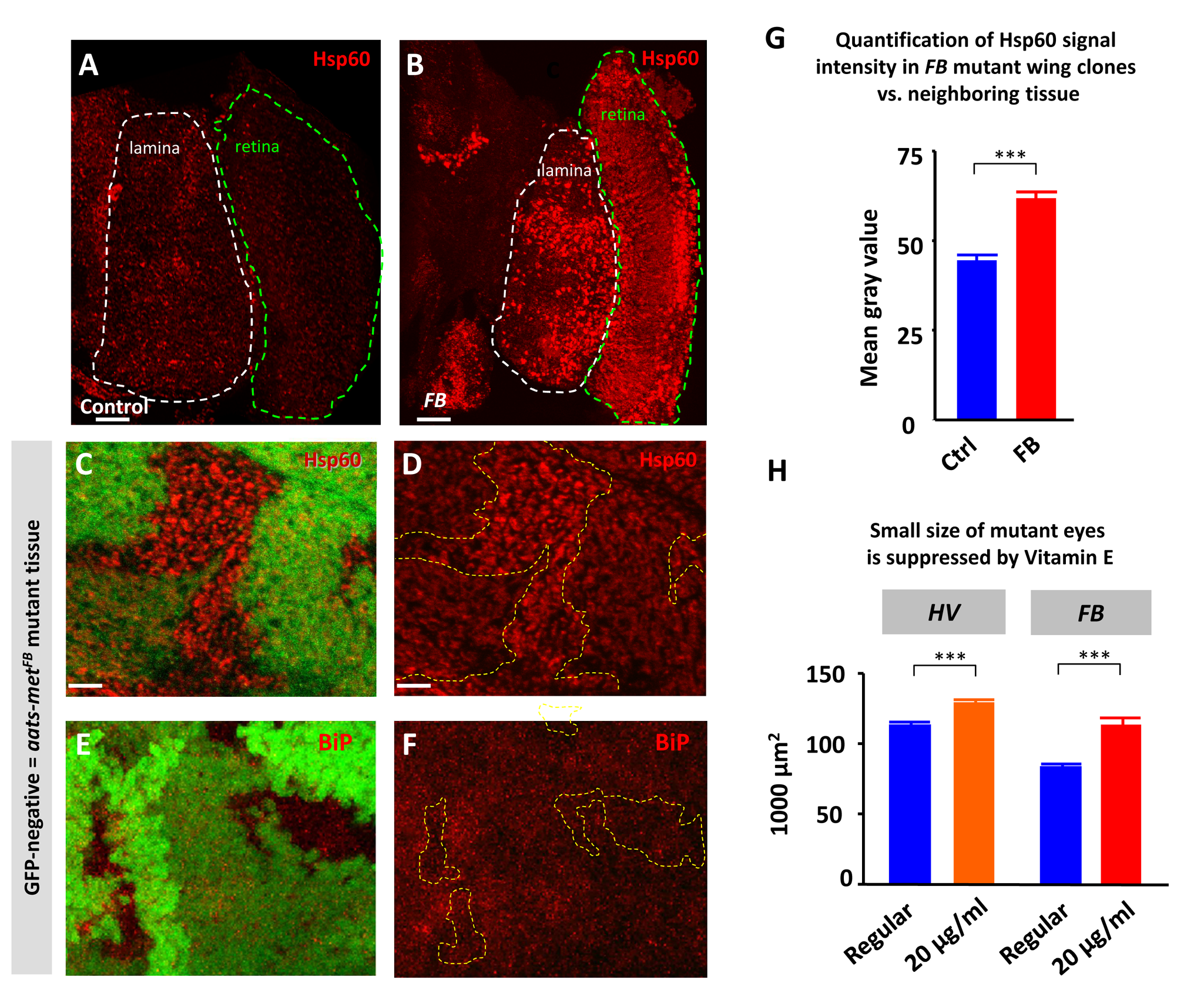

Supplement: Figure S3 — Upregulation of the mitochondrial unfolded protein response without concomitant cytoplasmic UPR response. (A) A control adult eye (y w eyFLP; FRT82B iso/FRT82B w+ cl) stained with anti-Hsp60, a protein that has been implicated as a marker of the UPRmt. (B) A mutant eye (y w eyFLP; FRT82B Aats-metFB/FRT82B w+ cl) stained with anti-Hsp60 shows a marked increase in staining in the retina and lamina (where the flippase is expressed). The dashed white lines mark the lamina, and the green lines mark the retina. (C–D) Heat-shock clones of Aats-metFB were generated in the wing imaginal disc (negatively marked for GFP) and stained for anti-Hsp60 (red), showing elevated levels of Hsp60 in mutant clones. Genotype: y w hsFLP; FRT82B Aats-metFB/FRT82B Ubi-GFPNLS. (E–F) Similar experiments were done with anti-BiP, a marker of the cytoplasmic UPR, showing unchanged levels in Aats-metFB mutant clones. (G) Quantification of the increased levels of Hsp60 in mutant clones versus neighboring tissue. (H) Quantification of the eye surface area of eyes carrying HV and FB mutant clones, untreated and treated with 20 µg Vitamin E, showing that it suppresses the small eye phenotype. (TIF) [file pbio.1001288.s003.tif]

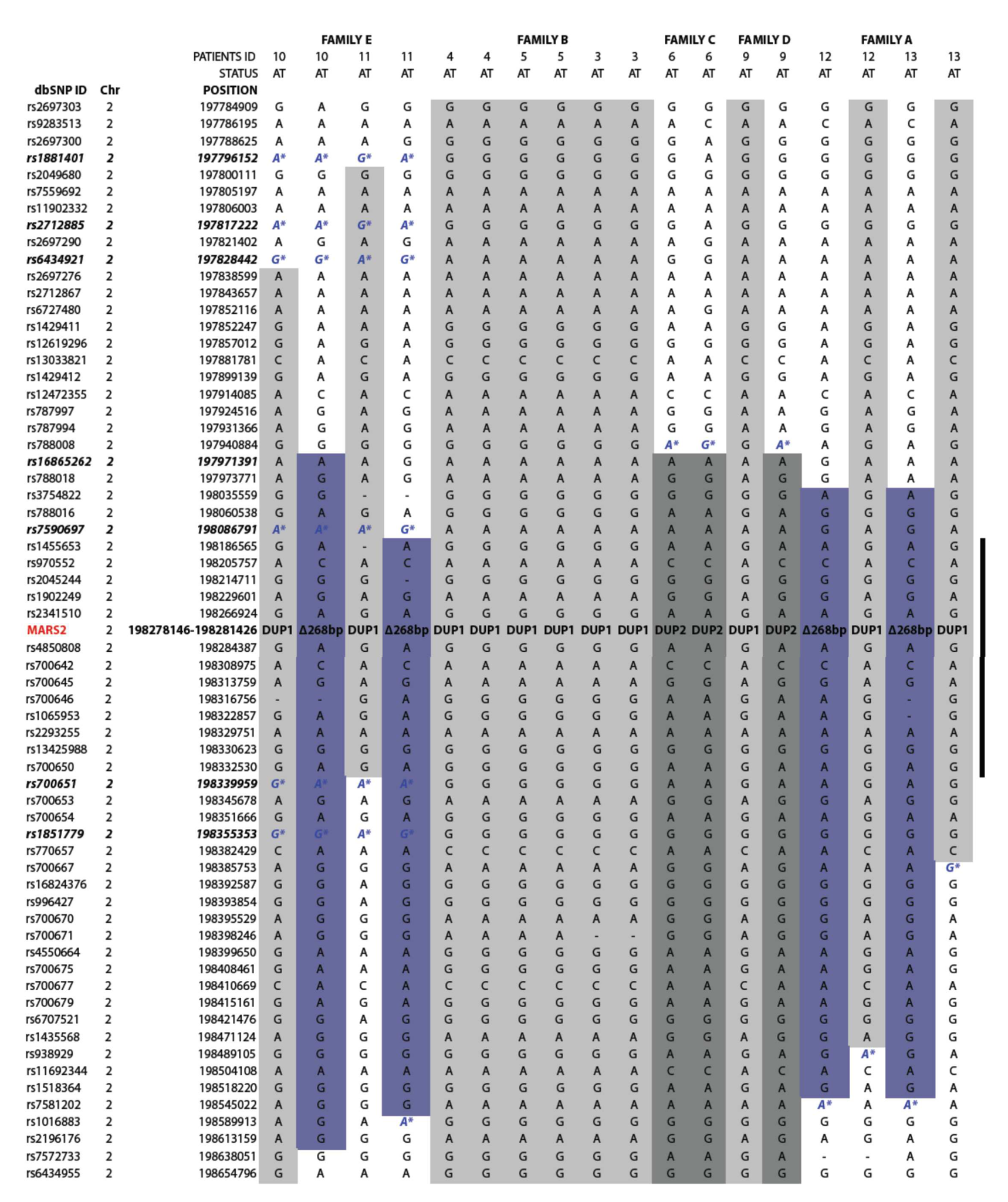

Supplement: Figure S4 — Homozygosity mapping by SNP microarray analysis. Homozygosity and haplotype analysis of DNA samples from nine patients belonging to five ARSAL families. Homozygosity spans over 50 Mb in Family B (unpublished data). Three common haplotypes on chromosome 2q33–34 surrounding the MARS2 region were identified (indicated in light grey for Dup1, dark grey for Dup2, and blue for the Dup-Del). An overlapping region for the three haplotypes was identified (black bar). (TIF) [file pbio.1001288.s004.tif]

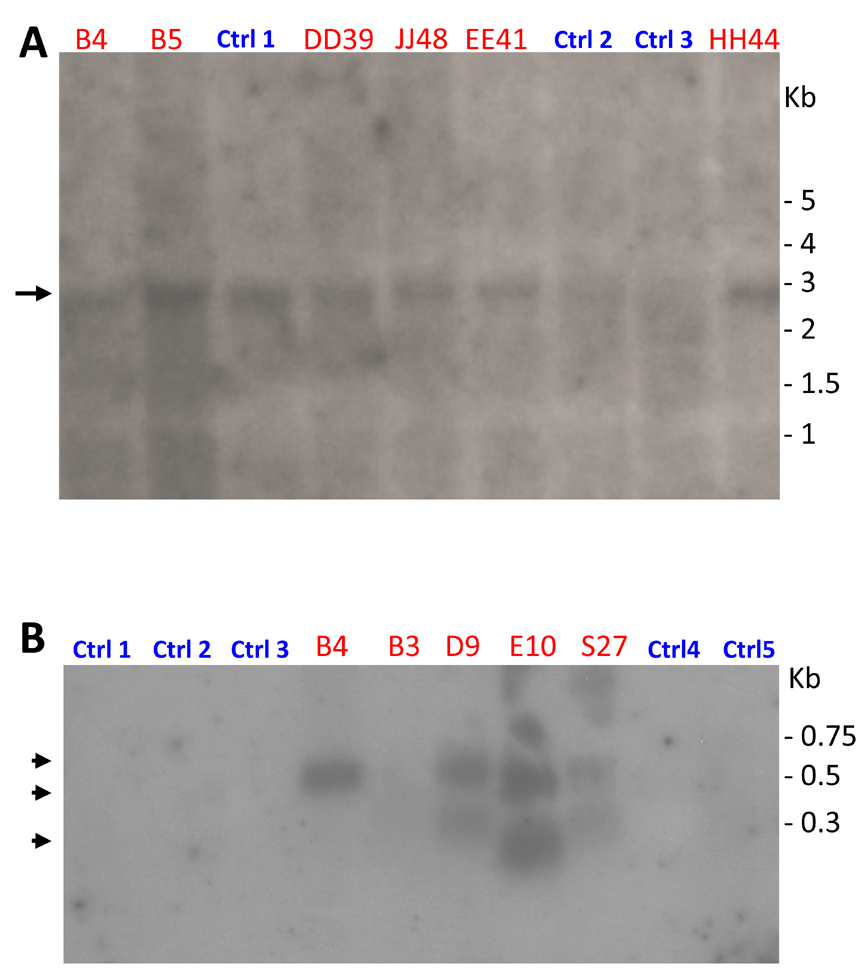

Supplement: Figure S5 — Northern blots of ARSAL patients. (A) Northern blot of six patients' and three controls' lymphoblasts is displayed. mRNAs of the same size (arrow) were detected by using a cDNA probe covering the entire MARS2 coding sequence for all cases examined. (B) Northern blot of patients' and controls' lymphoblasts is displayed. mRNA degradation (arrowheads) was detected using a cDNA probe covering 875 bp MARS2 coding sequence for all patients examined but not in the controls. Red lettering indicates patients and blue lettering refers to controls. (TIF) [file pbio.1001288.s005.tif]

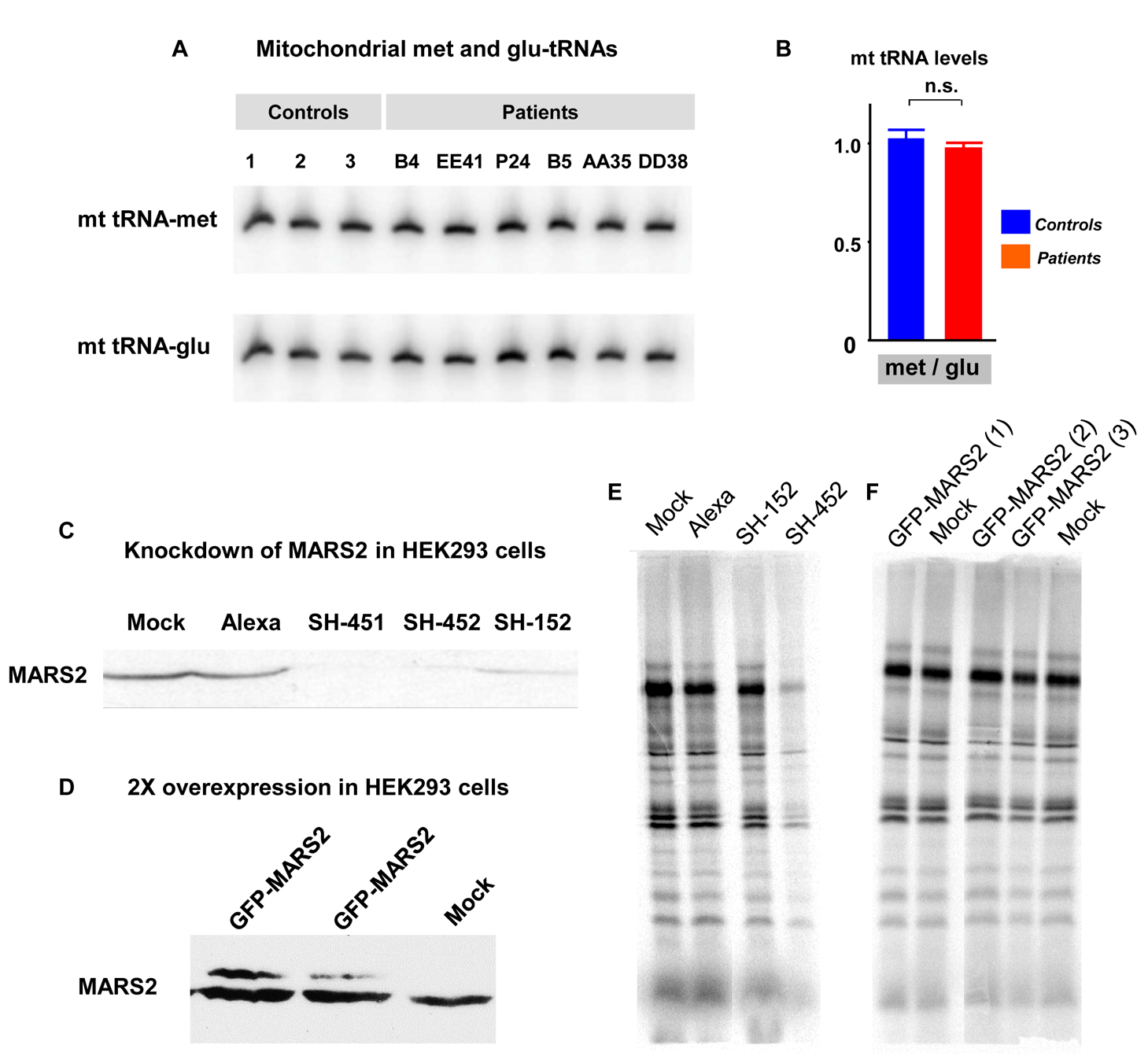

Supplement: Figure S6 — Mitochondrial tRNAs are stable in ARSAL patient cells, and loss of MARS2 in cells results in impaired mitochondrial translation. (A) Total steady-state levels of mitochondrial (mt) tRNA-met in patients and controls are similar, suggesting that decreased amino-acylation does not interfere with the stability of mt tRNA-met. mt tRNA-glu was used as a loading control. (B) Quantification of the mitochondrial methionyl-tRNA level relative to mitochondrial glutamic acid-tRNA is shown. MARS2 protein levels and mitochondrial protein translation. (C) Western blot of MARS2 protein performed on HEK293 cells transfected with shRNA constructs against human MARS2. Relative expression levels were normalized to prohibitin levels and the two controls (Mock, Alexa). shRNA constructs reduce MARS2 protein levels (SH-451: 15% of control, SH-452: 25%, SH-152: 75%). (D) Western blot of MARS2 protein in HEK293 cells expressing a MARS2-GFP transgene that results in 2× normal expression. (E) Mitochondrial protein synthesis was measured in siRNA experiments by pulse-labeling mitochondrial translation products with 35S-methionine for 1 h in the presence of emetine, followed by electrophoresis on a 15%–20% linear-gradient polyacrylamide gel. The 13 mitochondrial products are evident. A significant generalized mitochondrial translation deficiency is observed when the protein level of MARS2 is reduced to 25% of controls. There was too much cell death caused by SH-451 expression to perform the translation assay. (F) Mitochondrial protein synthesis was measured after GFP-MARS2 overexpression in HEK293 cells by pulse-labeling mitochondrial translation products with 35S-methionine for 1 h in the presence of emetine, followed by electrophoresis. The experiment was conducted 3 times. No impact on mitochondrial translation is observed. (TIF) [file pbio.1001288.s006.tif]

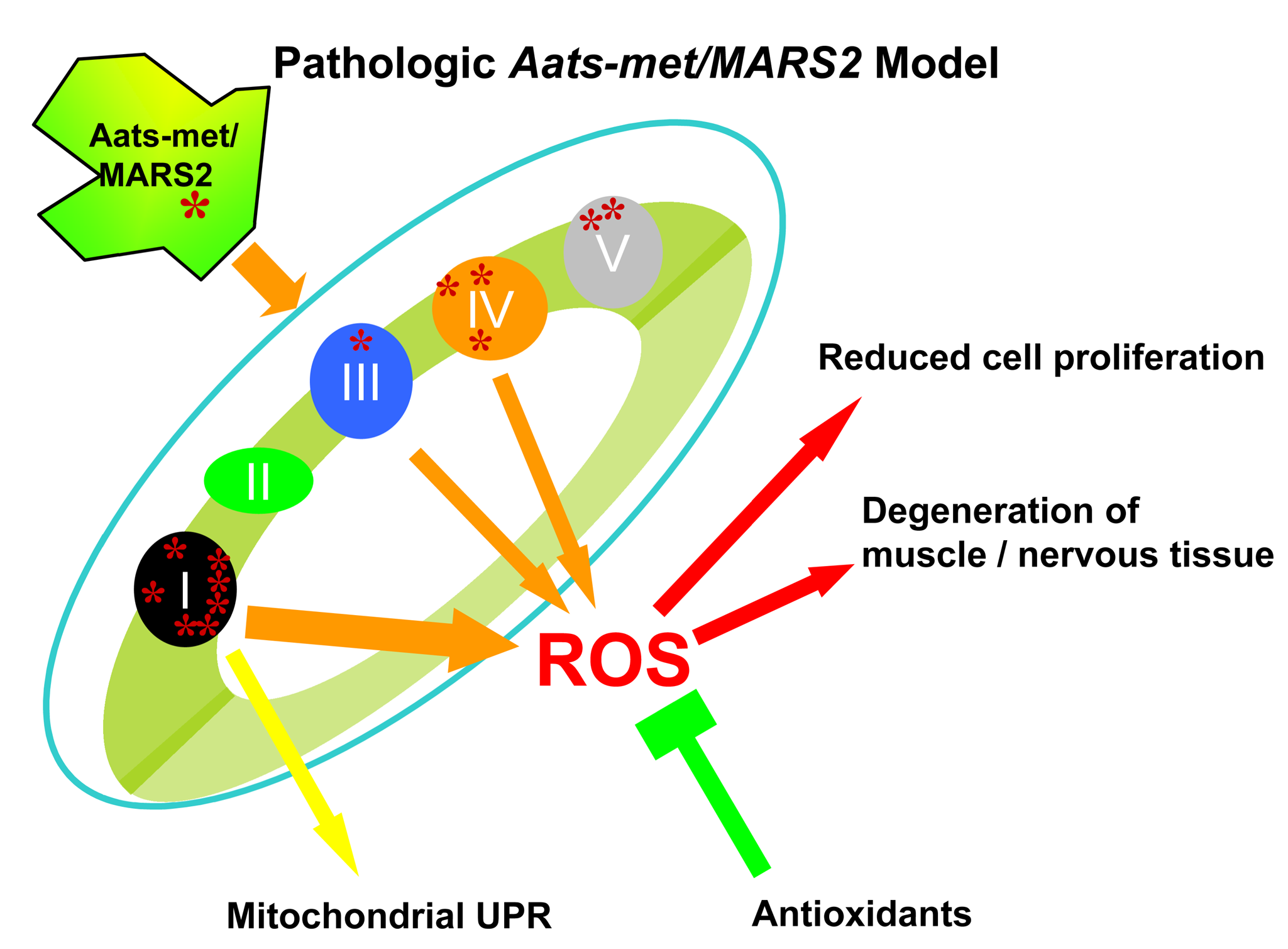

Supplement: Figure S7 — Pathologic Aats-met model. The model can be summarized as follows. Mutations in Aats-met result in impaired translation of the 13 components of Complexes I, III, IV, and V that are encoded in the mitochondrial genome. This results in impaired complex formation, a mitochondrial UPR, and an uncoupled respiratory chain. The resulting ROS causes tissues to degenerate, most notably neurons and muscle, and also affects cell proliferation via its effect on the cell cycle (JNK signaling). These effects of degeneration and cell proliferation can be partially suppressed by antioxidant supplementation. (TIF) [file pbio.1001288.s007.tif]
