# Supplementary material for: Mutations in the Mitochondrial Methionyl-tRNA Synthetase Cause a Neurodegenerative Phenotype in Flies and a Recessive Ataxia (ARSAL) in Humans
Source: PLoS Biol. 2012 Mar 20;10(3):e1001288. doi: 10.1371/journal.pbio.1001288 (PMC3308940; doi:10.1371/journal.pbio.1001288)
Supplement: Table S1 — Respiration rates of isolated mitochondria from control and Aats-met mutant larvae. The respiration rates for isolated mitochondria from 3rd instar larvae are listed for each of the genotypes used—control (FRT82B isogenized), HV/Df, and FB/Df, with means and standard deviations listed. (PDF) [file pbio.1001288.s008.pdf]

Table S1. Respiration Rates of Isolated Mitochondria from Control and *Aats-met* Mutant Larvae

	Glutamate+Malate[a]									Succinate[a]								
	III[b]			IV[c]			UC[d]			III			IV			UC		
Genotype	Mean	SD	N	Mean	SD	N	Mean	SD	N	Mean	SD	N	Mean	SD	N	Mean	SD	N
Control	56.95	21.00	6	27.80	11.02	6	62.76	23.62	6	62.84	15.72	6	27.61	7.42	6	76.19	30.35	6
HV/Def	52.76	20.63	6	27.78	9.20	6	39.50	19.15	6	89.74	36.31	6	36.95	9.79	6	75.82	32.77	6
FB/Def	50.25	15.55	6	33.59	10.36	6	36.95	17.63	6	127.87	58.05	6	56.25	22.90	6	112.25	46.04	6

[a]Respiration rates expressed as ng atomic O/min/mg mitochondrial protein
[b]III = State III (ADP-stimulated) respiration
[c]IV = State IV (ADP-independent) respiration
[d]UC = Uncoupled respiration
