# Supplementary material for: Mutations in the Mitochondrial Methionyl-tRNA Synthetase Cause a Neurodegenerative Phenotype in Flies and a Recessive Ataxia (ARSAL) in Humans
Source: PLoS Biol. 2012 Mar 20;10(3):e1001288. doi: 10.1371/journal.pbio.1001288 (PMC3308940; doi:10.1371/journal.pbio.1001288)
Supplement: Table S2 — Respiratory chain enzyme activities of isolated sonicated mitochondria from control and Aats-met mutant larvae. The enzyme activities, with means and standard deviations, for Complexes I, II, III, IV, and Citrate Synthase are listed. The genotypes used were control (FRT82B isogenized), HV/Df, and FB/Df. (PDF) [file pbio.1001288.s009.pdf]

Table S2. Respiratory Chain Enzyme Activities of Isolated Sonicated Mitochondria from Control and *Aats-met* Mutant Larvae

	Complex I[a]			Complex II[b]			Complex III[c]			Complex IV[d]			Citrate Synthase[e]		
**Genotype[¶]**	**Mean**	**SD**	**N**	**Mean**	**SD**	**N**	**Mean**	**SD**	**N**	**Mean**	**SD**	**N**	**Mean**	**SD**	**N**
**Control**	140.62	33.33	6	85.92	12.43	6	34.85	3.41	6	68.18	13.88	6	194.37	21.15	6
HV/Def	72.68	11.87	6	65.17	23.38	6	43.28	8.79	6	64.47	18.57	6	258.02	35.29	6
FB/Def	92.43	24.16	6	81.77	23.05	6	31.93	7.63	6	72.45	29.48	6	228.31	25.07	6

[a]NADH:Ubiquinol Oxidoreductase activity in nmol/min/mg protein
[b]Succinate Dehydrogenase activity in nmol/min/mg protein
[c]Ubiquinol:Cytochrome c Oxidoreductase activity in nmol/min/mg protein
[d]Cytochrome c Oxidase activity in nmol/min/mg protein
[e]nmol/min/mg protein
