# Supplementary material for: Mutations in the Mitochondrial Methionyl-tRNA Synthetase Cause a Neurodegenerative Phenotype in Flies and a Recessive Ataxia (ARSAL) in Humans
Source: PLoS Biol. 2012 Mar 20;10(3):e1001288. doi: 10.1371/journal.pbio.1001288 (PMC3308940; doi:10.1371/journal.pbio.1001288)
Supplement: Table S3 — Drosophila and human mitochondrially encoded proteins possess many methionines. The Drosophila and human mitochondrially encoded proteins are listed in the first column. The Respiratory Complex that they each belong to is listed in the second column. The number of methionines and methionine percentage of the Drosophila proteins is listed in the third column. The number of methionines and methionine percentage of the human proteins is listed in the fourth column. (PDF) [file pbio.1001288.s010.pdf]

Table S3

**Protein**	**Complex**	**Methionines *D. melanogaster***	**Methionines *H. sapiens***
**ND1**	**Complex I**	**13 / 312 (4.17%)**	**16 / 318 (5.03%)**
**ND2**	**Complex I**	**32 / 341 (9.38%)**	**24 / 346 (6.94%)**
**ND3**	**Complex I**	**6 / 117 (5.13%)**	**8 / 115 (6.96%)**
**ND4**	**Complex I**	**33 / 446 (7.40%)**	**27 / 459 (5.88%)**
**ND4L**	**Complex I**	**11 / 96 (11.46%)**	**10 / 98 (10.20%)**
**ND5**	**Complex I**	**51 / 574 (8.89%)**	**26 / 603 (4.31%)**
**ND6**	**Complex I**	**16 / 174 (9.20%)**	**10 / 174 (5.75%)**
**CytB**	**Complex III**	**12 / 378 (3.17%)**	**15 / 380 (3.95%)**
**Cox1**	**Complex IV**	**23 / 511 (4.50%)**	**32 / 513 (6.24%)**
**Cox2**	**Complex IV**	**10 / 228 (4.39%)**	**10 / 227 (4.41%)**
**Cox3**	**Complex IV**	**7 / 262 (2.67%)**	**11 / 261 (4.21%)**
**ATP6**	**Complex V**	**16 / 224 (7.14%)**	**12 / 226 (5.31%)**
**ATP8**	**Complex V**	**4 / 53 (7.55%)**	**6 / 68 (8.82%)**
