# Supplementary material for: Mutations in the Mitochondrial Methionyl-tRNA Synthetase Cause a Neurodegenerative Phenotype in Flies and a Recessive Ataxia (ARSAL) in Humans
Source: PLoS Biol. 2012 Mar 20;10(3):e1001288. doi: 10.1371/journal.pbio.1001288 (PMC3308940; doi:10.1371/journal.pbio.1001288)
Supplement: Table S4 — Primers used. The primers used for quantitative PCR, sequencing of the MARS2 genomic region and cDNA, and for the CNV assays are displayed. (PDF) [file pbio.1001288.s011.pdf]

Table S4. ARSAL primers and ABI®-based Copy Number Assays for human copy number screening

ARSAL qPCR Primers		
**PRIMERS**	**SEQUENCES**	**PRODUCT SIZE (bp)**
GAPDH-f	GGTGCCTGATATAGCTTGAC	121
GAPDH-r	GCTGGATGACGTGAGTAAAC	
MARS2.q.F1	GCCAGGGTTGGTGGGGC	119
MARS2.q.R1	CCTTATATATCCGAAAGTTATCATAGTGGTCTGCT	
MARS2.q.F2	TCTCCTGCACAGATTTCATCCGCA	121
MARS2.q.R2	CAATACCAACCTTCATAGACGCCCTTG	
MARS2.q.F3	TGAGGGTCAGGGTTGGAGGC	158
MARS2.q.R3	TTTAAATGGGGATACACATGGGCAAAAATCA	
BC021693_q1-F	AATTCCAGTATGCTGCAGTGG	205
BC021693_q1-R	TGCAAGGGTCAATGTTATGAG	
BC021693_q1-2F	CGGGTGTTGGTAATGGAG	269
BC021693_q1-2R	TGTAGGAAGATTACTATGTTCAGCC	
HSP60_F (HSPD1)	GAGTAGAGGCGGAGGGAGG	121
HSP60_R (HSPD1)	CGTTTGCAGAAATCGTAGCA	
PLCL1_F	CCTGAGCAAAAGAAGGTTGC	119
PLCL1_R	GGAGGTAGGATTCTGAGGGC	

ARSAL cDNA Primers		
**PRIMERS**	**SEQUENCES**	**PRODUCT SIZE (BP)**
MARS2_Cdna_1F	CACCATGCTGCGAACGTC	284
MARS2_Cdna_1R	CGTCGGTACCAGTGGAGAAT	
MARS2_Cdna_2F	CTACTGGCGGACGCCCTAT	294
MARS2_Cdna_2R	GACGCCCTTGTAGAGCAGAC	
MARS2_Cdna_3F	ACAGATTTCATCCGCACCAC	386
MARS2_Cdna_3R	TGGCTACTTCTGCGAGACAC	
MARS2_Cdna_4F	CCGAACCATTTCATCACGTA	380
MARS2_Cdna_4R	GCAAGTCCTAGGATCCACCA	
MARS2_Cdna_5F	CCATTCCCACTGGACAGTCT	553
MARS2_Cdna_5F	CCCAAAGACTCGCAAACATT	
MARS2cDNA synthesis-f	GATGATGCTTGTGATGCGCGCCTAC	1668
MARS2cDNA synthesis-r	GGTCCGGTGGGCTTTCACCAGCCAAG	
MARS2cDNA_ 5UTR-f	CACCATGCTGCGAACGTC	2934
MARS2cDNA_3UTR-r	CAGTTAAAATTACCTGAAAGTTGTGT	
BC021693_cDNAf	GCCTTGCAGTTTGATCTCAG	951
BC021693_cDNAr	AGGAGACAATCAAAGTATCCTCAG	

ARSAL Genomic Primers		
**PRIMERS**	**SEQUENCES**	**EXON & PRODUCT SIZE (bp)**
MARS2_5utr-gc-RPT-f	TACGAAGGCAAAGAGGTTCG	380
MARS2_5utr-gc-RPT-r	CCTCCAGGAGAGACAGCCTA	
MARS2_(gcn)RPT-f	CGAGCTGTTTTTCTGGCAAGC	221
MARS2_(gcn)RPT-R	TGACAGCACGTGTGAGAGG	
Mars2_EXON1_A	CGTTTCATTGGCTTTCCG	886
Mars2_EXON1_B	TACTTCTGCGAGACACGGAC	

Mars2_EXON1-2_A	AGTTCCGGAAGCCACTCC	875
Mars2_EXON1-2_B	AGTACCCAGCCAGGGAGC	
Mars2_EXON1-3_A	ATATAAGGCTCTGGAGGCCG	874
Mars2_EXON1-3_B	TCTAGGACACTGGTTCAAGGG	
Mars2_EXON1-4_A	TTACTGGTGGTTTTACAGGGG	871
Mars2_EXON1-4_B	AAGCATTGGTCAGTTCCTCC	
Mars2_promoteur_a	TGATCACACAAATGTGCAAGAA	656
Mars2_promoteur_b	CAACAAAAGTAGATTTGTGATCTGC	
Mars2_promoteur_2a	TTGGCAAGAACTTGGCAGAT	787
Mars2_promoteur_2b	GCACATCACAAGCATCATCG	
bc021684_EX1a	CACTTGAGGAGGGAGTCTGC	459
bc021684_EX1b	CCAGTCAAAGAAAGGGGTGA	
bc021684_EX2a	ATTTGGGACCTCCCATGTTA	488
bc021684_EX2b	TGACAAGTTAATGGGTGCAG	
bc021684_EX3a	TGTGGCAAAATGTTGATGTCT	396
bc021684_EX3b	TTCAATTTTTACCATAGCTACATAGG	
bc021684_EX4a	ACTCTGGCTGCCTTGTGAAT	1194
bc021684_EX4b	TTTTCCCTTAGCCACATCCT	
BC021693_EP_ex3A	AAGAGGGTAAGGAAGTGGGG	Exon3: 58 bp, Product size 651
BC021693_EP_ex3B	TTTGTGTAGCATACTGGTGTTGG	
BC021693_EP_ex4A	CGGGTGTTGGTAATGGAG	Exon4: 809 bp, Product size 1000
BC021693_EP_ex4B	TTGTATTCATTTGGACTGTTATGC	
Mars_ex1re-A	GAGCTGTTTTTCTGGCAAGC	862
Mars_ex1re-B	TTCCTTGGTCCAGGAGACTG	
Mars_ex2re-A	GGCACCTGTACTCGGCACTA	902
Mars_ex2re-B	GCAAGTCCTAGGATCCACCA	
Mars_ex2re-C	GGGGGATTCGTTTCCTGTAT	997
Mars_ex2re-D	CCCAAAGACTCGCAAACATT	
Mars_ex3re-A	TCTCTTGAACCGATGCACTG	806
Mars_ex3re-B	CCATCTTTCCCCTGAAGACA	
MARS2_LR_FR1-F	GGGTTTAGACAAACGGCTGA	2461
MARS2_LR_FR1-R	TGGGCCCTCTCTACTTGCTA	
MARS2_LR_FR2-F	GGGTTTAGACAAACGGCTGA	1699
MARS2_LR_FR2-R	AGGGAAGCAGGTAGTGCAGA	

ARSAL CNV ASSAYS	CNV ASSAY_ID	PROBE POSITIONS & INTERSPACE (bp)		EXON
MARS2	Hs02277482_cn	198278558		1
	Hs02141409_cn	198279333	775	1
	Hs01848862_cn	198279929	596	1
	Hs02550138_cn	198280672	743	1
HSPD1	Hs00304988_cn	198062204	216354	4
COQ10	Hs00506215_cn	198043117	235441	4
PLCL1	Hs01068343_cn	198657233	378675	3
