# Supplementary material for: Mutations in the Mitochondrial Methionyl-tRNA Synthetase Cause a Neurodegenerative Phenotype in Flies and a Recessive Ataxia (ARSAL) in Humans
Source: PLoS Biol. 2012 Mar 20;10(3):e1001288. doi: 10.1371/journal.pbio.1001288 (PMC3308940; doi:10.1371/journal.pbio.1001288)
Supplement: Table S5 — ARSAL patients' genetic variations and clinical characteristics. ARSAL patients are listed. Alongside them are their family identifiers, gender, their genetic variations, the method by which their mutations were identified, the age of symptom onset, and the presence or absence of 11 clinical/imaging characteristics. (PDF) [file pbio.1001288.s012.pdf]

Table S5. Major clinical features and MARS2 mutations in French Canadian individuals with ARSAL

Patient no.	Gender	Family	Mutation 1	Mutation 2	Mutation detection	Age of onset	Ataxia	Spasticity	Dysarthria	Dysmetria	Nystagmus	Dystonia	Scoliosis	U. urgency	Wheelchair use, y	leukodystrophy	Cerebellar atrophy	
																	Vermis	Hemispheres
10	M	E	Dup1	c.681∆268bpfx236X	PCR, Sequencing, CNV assays, CGH-array, Western Blot	15	•	•	•		•	•				+	++	++
11	M	E	Dup1	c.681∆268bpfx236X	PCR, Sequencing, CNV assays, Western Blot	3	•	•	•		•	•						
3	F	B	Dup1	Dup1	PCR, Sequencing, CNV assays, CGH-array, Western Blot	3	•	•	•	•	•	•	•	•	51	+	+	+
4	M	B	Dup1	Dup1	PCR, Sequencing, CNV assays, CGH-array, Western Blot	6	•	•	•	•	•	•	•		19	+	+++	+++
5	M	B	Dup1	Dup1	PCR, Sequencing, CNV assays, Western Blot	3	•	•			•	•	•		45	+	++	+
6	M	C	Dup2	Dup2	PCR, Sequencing, CNV assays, Western Blot	20	•	•	•	•	•	•		•	59	+	++	++
8	F	C	Dup2	Dup2	PCR, Sequencing, CNV assays, Western Blot	17	•	•	•	•	•					-	++	++
1	F	C	Dup2	Dup2	PCR, Sequencing, CNV assays	47	•	•		•		•		•				
2	M	C	Dup2	Dup2	PCR, Sequencing, CNV assays	50	•	•	•			•		•				
9	F	D	Dup1	c.681∆268bpfx236X	PCR, Sequencing, CNV assays, Western Blot	13	•	•	•		•	•		•	30	-	+	+
12	F	A	Dup1	c.681∆268bpfx236X	PCR, Sequencing, CNV assays	3	•	•	•			•	•	•	10	-	++	++
13	F	A	Dup1	c.681∆268bpfx236X	PCR, Sequencing, CNV assays, CGH-array	3	•	•	•					•		-	+++	+++
14	M	F	Dup1	Dup1	PCR, Sequencing, CNV assays	5	•	•								+	+	-
15	F	G	Dup1	Dup1	PCR, Sequencing, CNV assays	3	•	•	•	•	•		•		45			
7	M	H	Dup1	Dup1	PCR, Sequencing, CNV assays	19	•	•	•	•	•					-	+++	+++
17	M	I	Dup1	Dup1	PCR, Sequencing, CNV assays	23	•	•				•		•				
18	M	I	Dup1	Dup1	PCR, Sequencing, CNV assays	30	•	•	•	•	•				38	-	+++	++
16	F	J	Dup1	Dup2	PCR, Sequencing, CNV assays	2	•	•		•		•	•	•	36	-	+	-
21	F	K	Dup1	Dup1	PCR, Sequencing, CNV assays	59	•	•					•		72	-	+	+
18	F	L	Dup1	Dup2	PCR, Sequencing, CNV assays	43	•	•	•	•						+	++	++
19	M	M	Dup1	Dup2	PCR, Sequencing, CNV assays	26	•	•		•		•			38	+	++	++
20	M	N	Dup1	Dup2	PCR, Sequencing, CNV assays	4	•	•	•	•				•		-	+++	+++
24	M	P	Dup1	Dup1	PCR, Sequencing, CNV assays, Western Blot	9	•	•	•	•	•			•	16	+	+	-
26	F	R	Dup1	Dup1	PCR, Sequencing, CNV assays	2	•	•	•				•			-	+++	+++
28	F	T	Dup1	Dup1	PCR, Sequencing, CNV assays	32	•	•	•	•		•		•		+	+++	+++
29	M	U	Dup1	Dup1	PCR, Sequencing, CNV assays	48	•	•			•			•		-	+	+
30	F	X	Dup1	Dup1	PCR, Sequencing, CNV assays	50	•	•	•	•						-	+	-
31	M	Y	Dup1	c.681∆268bpfx236X	PCR, Sequencing, CNV assays	5	•	•	•	•					14			
32	M	Z	Dup1	Dup1	PCR, Sequencing, CNV assays	13	•	•	•	•						-	+++	+++
33	F	Z	Dup1	Dup1	PCR, Sequencing, CNV assays	48	•	•	•		•					+		
34	M	AA	Dup1	Dup2	PCR, Sequencing, CNV assays	29	•	•	•	•	•			•				
35	F	AA	Dup1	Dup2	PCR, Sequencing, CNV assays, Western Blot	26	•	•	•	•	•					-	++	++
36	M	BB	Dup1	c.681∆268bpfx236X	PCR, Sequencing, CNV assays	16			•									
37	M	CC	Dup1	Dup1	PCR, Sequencing, CNV assays	28	•	•	•	•					53	-	+++	++
38	M	DD	Dup1	Dup2	PCR, Sequencing, CNV assays	36	•	•	•		•			•				
39	M	DD	Dup1	Dup2	PCR, Sequencing, CNV assays, Western Blot	40	•	•	•	•	•			•		-	-	-
40	F	DD	Dup1	Dup2	PCR, Sequencing, CNV assays, Western Blot	35	•	•			•			•		-	++	++
41	M	EE	Dup1	Dup1	PCR, Sequencing, CNV assays, Western Blot	25	•	•	•	•				•		-	-	-
42	M	FF	Dup1	Dup1	PCR, Sequencing, CNV assays	30	•	•	•	•	•			•		-	+	+
43	F	GG	Dup2	Dup2	PCR, Sequencing, CNV assays	30	•	•	•		•					+	++	++
44	F	HH	Dup1	c.681∆268bpfx236X	PCR, Sequencing, CNV assays, Western Blot	13	•	•	•			•		•		+	+	-
45	M	HH	Dup1	c.681∆268bpfx236X	PCR, Sequencing, CNV assays, Western Blot	14	•	•	•							+	-	-
46	M	II	Dup2	Dup2	PCR, Sequencing, CNV assays	32	•	•	•	•				•				
47	M	II	Dup2	Dup2	PCR, Sequencing, CNV assays	44	•	•	•	•	•			•				
48	M	JJ	Dup1	Dup1	PCR, Sequencing, CNV assays	41	•	•	•	•				•		+	-	-
49	F	KK	Dup1	Dup2	PCR, Sequencing, CNV assays	37	•	•	•	•				•		-	+++	++
50	M	KK	Dup1	Dup2	PCR, Sequencing, CNV assays	20	•	•	•	•				•	42	-	+++	+++
51	F	KK	Dup1	Dup2	PCR, Sequencing, CNV assays	38	•	•	•	•						+	+	+
52	M	LL	Dup1	Dup1	PCR, Sequencing, CNV assays	13	•	•			•		•			+	+	+
53	F	MM	Dup1	Dup1	PCR, Sequencing, CNV assays	40	•	•	•	•						-	+++	+++
54	M	NN	Dup1	Dup2	PCR, Sequencing, CNV assays	40	•	•	•	•					51	+	+++	-
55	F	OO	Dup1	Dup1	PCR, Sequencing, CNV assays	45	•	•	•					•	59	-	++	++
56	F	PP	Dup1	Dup1	PCR, Sequencing, CNV assays	2	•	•	•							-	+++	-
57	F	RR	Dup1	Dup1	PCR, Sequencing, CNV assays	40	•	•			•			•		-	+	+
Mean/%						24.4	100%	100%	78%	57%	44%	28%	17%	48%	39.9	40%	93%	75%

Legend:

Dup1: Duplication 1: Dup2: Duplication 2; U. urgency: urinary urgency; y: year; CNV: Copy number variation; CGH: copy genomic hybridization
