# Supplementary material for: Mutations in the Mitochondrial Methionyl-tRNA Synthetase Cause a Neurodegenerative Phenotype in Flies and a Recessive Ataxia (ARSAL) in Humans
Source: PLoS Biol. 2012 Mar 20;10(3):e1001288. doi: 10.1371/journal.pbio.1001288 (PMC3308940; doi:10.1371/journal.pbio.1001288)
Supplement: Table S6 — Mitochondrial protein synthesis. Quantification of mitochondrial protein synthesis shows a generalized deficiency in the patients homozygous for the common mutation (54%, 67%, and 79% of the average of controls). On the other hand, patients who are compound heterozygous for MARS2 mutations have normal mitochondrial translation (89%, 107%, and 118% of the average of controls). (PDF) [file pbio.1001288.s013.pdf]

Table S6. Quantification of mitochondrial protein synthesis

	C1	C2	C3	DD38	B4	E10	P24	B5	AA35
**%**	**80**	**97**	**105**	**118**	**54**	**89**	**67**	**79**	**107**
**ND5**	105	91	99	105	32	75	50	28	103
**CO I**	80	98	104	119	54	96	71	69	111
**ND4**	97	120	79	104	22	67	58	75	102
**Cyt b**	71	99	98	133	57	74	45	115	86
**ND2**	77	90	111	122	49	79	53	88	113
**ND1**	45	74	98	182	36	81	31	87	115
**CO III**	71	104	96	128	57	84	60	81	103
**CO II**	74	91	109	126	55	80	66	84	97
**ATP6**	98	112	88	102	51	119	83	95	107
**ND6**	96	104	116	84	60	90	100	80	116
**ND3**	85	89	127	100	52	82	78	61	91
**A8/ND4L**	87	96	128	89	90	117	97	101	118
