# Supplementary material for: Mutations in the Mitochondrial Methionyl-tRNA Synthetase Cause a Neurodegenerative Phenotype in Flies and a Recessive Ataxia (ARSAL) in Humans
Source: PLoS Biol. 2012 Mar 20;10(3):e1001288. doi: 10.1371/journal.pbio.1001288 (PMC3308940; doi:10.1371/journal.pbio.1001288)
Supplement: Table S7 — AARS diseases. These 12 AARS-related diseases, the responsible genes, and their documented clinical phenotypes are listed. Note that those genes with a “2” at the end of their name (i.e., MARS2, DARS2, RARS2, YARS2, HARS2, AARS2, SARS2, and LARS2) are purely mitochondrial tRNA synthetases. GARS and KARS encode both the mitochondrial and cytoplasmic tRNA synthetases based on the splice forms translated, and YARS and AARS encode purely cytoplasmic synthetases. (PDF) [file pbio.1001288.s014.pdf]

Table S7. Major clinical features of patients with AARS diseases

Human Disease	Ataxia with leukoencephalopathy [ARSAL]	Leukoencephalopathy [LBSL]	Pontocerebellar Hypoplasia [PCH6]	Myopathy, Lactic Acidosis, and Sideroblastic Anemia [MLASA]	Charcot-Marie-Tooth disease type 2 [CMT2D] [CMT2D]	Charcot-Marie-Tooth disease type 2 [CMT2N]
	Recessive	Recessive	Recessive	Recessive	Dominant	Dominant
Chromosome	2q33	1q25.1	6q16.1	12q11.21	7p15	16q.21-23
Genes	*MARS2*	*DARS2*	*RARS2*	*YARS2*	*GARS*	*AARS*
Cell Localization	Mito.	Mito.	Mito.	Mito.	Mito./Cytoplasm	Cytoplasm
Age at diagnosis	2-59	childhood	3 days	Childhood	childhood	Adult
Lactate elevation	Unknown	Yes	CSF lactate	Yes	No	No
Dysphagia	No	Unknown	Unknown	Yes	No	No
Myopathy	No	No	No	Yes	Yes	Yes
Spasticity	yes	Yes	Yes	No	No	No
Ataxia	Yes	Yes	Unknown	No	No	No
Dystonia	Yes	Yes	Yes	No	No	No
White matter changes	Yes	Yes	Yes	Unknown	No	No
Cerebellar atropy	Yes	Yes	Yes	Unknown	No	No
Corpus callosum atrophy	Yes	Yes	Yes	Unknown	No	No
Sensory involvement	No	Yes	Unknown	No	Yes	Yes
Neuropathy	No	No	No		Yes	Yes
Liver Disease	No	Unknown	Unknown	Unknown	No	No
Ovarian Dysgenesis	No	Unknown	Unknown	Unknown	No	No
Pulmonary Disease	No	Unknown	Unknown	Unknown	No	No
Renal Disease	No	Unknown	Unknown	Unknown	No	No
Diabetes						
Mental retardation	Yes	Yes	Yes	Yes	Rare	No
References:	Brain. 2006 Sep;129(Pt 9):2332-40. ; this manuscript	Nat Genet. 2007 Apr;39(4):534-9.	Am J Hum Genet. 2007 Oct;81(4):857-62.	Am J Hum Genet. 2010 Jul 9;87(1):52-9.	PNAS 2007 Jul 3;104(27):11239-44.	Am J Hum Genet. 2010 Jan 8;86(4):77-82.

Human Disease	Charcot-Marie-Tooth disease [DI CMT]	Intermediate Peripheral Neuropathy CMT-Like	Ovarian Dysgenesis and sensorineural hearing loss Perrault Syndrome	Infantile Mitochondrial Cardiomyopathy [CPM]	Hyperuricemia, pulmonary hypertension, renal failure in infancy and alkalosis HUPRA	Type 2 diabetes susceptibility gene
	Dominant	Dominant	Recessive	Recessive		Complex
Chromosome	1p34-35	16q23-1	5q31	6q21.1	19q13.2	3p21.3
Genes	*YARS*	KARS	*HARS2*	*AARS2*	*SARS2*	*LARS2*
Cell Localization	Cytoplasm	Mito./Cytoplasm	Mito.	Mito.	Mito.	Mito.
Age at diagnosis	Children-Adult	Children-Adult	Adult	Childhood	Childhood	Adult
Lactate elevation	No	No	Unknown	Yes	Yes	Unknown
Dysphagia	No	No	No	Yes	Unknown	No
Myopathy	Yes	Yes	No	Yes	No	No
Spasticity	No	No	No	Yes	No	Unknown
Ataxia	No	No	No	Unknown	No	Unknown
Dystonia	No	No	No	Unknown	Unknown	Unknown
White matter changes	No	No	Unknown	Unknown	Unknown	Unknown
Cerebellar atropy	No	No	Unknown	Unknown	Unknown	Unknown
Corpus callosum atrophy	No	No	Unknown	Unknown	Unknown	Unknown
Sensory involvement	Yes	Yes	Yes	Yes	Unknown	Unknown
Neuropathy	Yes	Yes	No	Yes	Unknown	Yes
Liver Disease	No	No	No	No	No	Unknown
Ovarian Dysgenesis	No	No	Yes	Unknown	Unknown	Unknown
Pulmonary Disease	No	No	No	Yes	Yes	Unknown
Renal Disease	No	No	No	No	Yes	Unknown
Diabetes					Yes	Yes
Mental retardation	No	Yes	No	Unknown	Yes	Unknown
References:	Mol Cell Neurosci. 2011 Feb;46(2):432-43.	Am J Hum Genet. 2010 Oct 8;87(4):560-6.	PNAS 2011 Apr 19;108(16):6543-8.	Am J Hum Genet. 2011 May 13;88(5):635-42.	Am J Hum Genet. 2011 Feb 11;88(2):193-200.	Diabetes. 2005 Jun;54(6):1892-5.
